# Pediatric CNS tumors and 2021 WHO classification: what do oncologists need from pathologists?

**DOI:** 10.3389/fnmol.2024.1268038

**Published:** 2024-03-13

**Authors:** Antonio d’Amati, Lavinia Bargiacchi, Sabrina Rossi, Andrea Carai, Luca Bertero, Valeria Barresi, Maria Elena Errico, Anna Maria Buccoliero, Sofia Asioli, Gianluca Marucci, Giada Del Baldo, Angela Mastronuzzi, Evelina Miele, Federica D’Antonio, Elisabetta Schiavello, Veronica Biassoni, Maura Massimino, Marco Gessi, Manila Antonelli, Francesca Gianno

**Affiliations:** ^1^Unit of Anatomical Pathology, Department of Precision and Regenerative Medicine and Ionian Area, University of Bari “Aldo Moro”, Bari, Italy; ^2^Unit of Human Anatomy and Histology, Department of Translational Biomedicine and Neuroscience (DiBraiN), University of Bari “Aldo Moro”, Bari, Italy; ^3^Unit of Anatomical Pathology, Department of Radiology, Oncology and Anatomical Pathology, University La Sapienza, Rome, Italy; ^4^Neuropathology Unit, Fondazione Policlinico Universitario "A. Gemelli" IRCCS, Università Cattolica S. Cuore, Roma, Italy; ^5^Pathology Unit, Department of Laboratories, Bambino Gesù Children’s Hospital, IRCCS, Rome, Italy; ^6^Department of Neuroscience and Neurorehabilitation, Bambino Gesù Children's Hospital, IRCCS, Rome, Italy; ^7^Pathology Unit, Department of Medical Sciences, University of Turin, Turin, Italy; ^8^Department of Diagnostics and Public Health, University of Verona, Verona, Italy; ^9^Department of Pathology, AORN Santobono Pausilipon, Pediatric Hospital, Naples, Italy; ^10^Pathology Unit, Meyer Children's Hospital IRCCS, Firenze, Italy; ^11^Department of Biomedical and Neuromotor Sciences (DIBINEM), Alma Mater Studiorum University of Bologna, Bologna, Italy; ^12^Neuropathology Unit, Fondazione IRCCS Istituto Neurologico Carlo Besta, Milan, Italy; ^13^Department of Paediatric Haematology/Oncology, IRCCS Bambino Gesù Children's Hospital, Rome, Italy; ^14^Pediatric Oncology Unit, Fondazione IRCCS Istituto Nazionale dei Tumori, Milan, Italy; ^15^IRCCS Neuromed, Pozzilli, Isernia, Italy

**Keywords:** pediatric CNS tumors, brain tumors, molecular biology, WHO classification, neuro-oncology, neuropathology

## Abstract

The fifth edition of the WHO Classification of Tumors of the Central Nervous System (CNS), published in 2021, established new approaches to both CNS tumor nomenclature and grading, emphasizing the importance of integrated diagnoses and layered reports. This edition increased the role of molecular diagnostics in CNS tumor classification while still relying on other established approaches such as histology and immunohistochemistry. Moreover, it introduced new tumor types and subtypes based on novel diagnostic technologies such as DNA methylome profiling. Over the past decade, molecular techniques identified numerous key genetic alterations in CSN tumors, with important implications regarding the understanding of pathogenesis but also for prognosis and the development and application of effective molecularly targeted therapies. This review summarizes the major changes in the 2021 fifth edition classification of pediatric CNS tumors, highlighting for each entity the molecular alterations and other information that are relevant for diagnostic, prognostic, or therapeutic purposes and that patients’ and oncologists’ need from a pathology report.

## 1 Introduction

The 2021 WHO Classification of Central Nervous System (WHO CNS5) is the fifth edition of the international standard for the classification of brain and spinal cord tumors. The WHO CNS5 combined the previous 2016 WHO classification with novel molecular pathogenic alterations that are fundamental for the most accurate classification of CNS neoplasms (Louis et al., 2021). The fifth edition changes relate mainly to pediatric CNS tumor classification, requiring an entire chapter for CNS pediatric tumors within the WHO Classification (Pfister et al., 2022). These changes included the integration of histologic diagnosis with molecular profile to formulate an integrated diagnosis; the introduction of novel molecular diagnostic techniques such as DNA methylation analysis for tumor classification and crucial diagnostic criterion, particularly for difficult-to-diagnose cases; the differentiation between “pediatric-type” and “adult-type” tumor categories, considering the different behaviors; the association with cancer-predisposition syndromes; and the identification of novel tumor entities (Torp et al., 2022). In the 2021 WHO classification, contrary to previously, CNS tumor grades are written using Arabic numerals, according to the classification of cancer in other organ systems and decreasing the mistakes of pathology report. In previous classification, CNS tumors received a grade assigned to each entity, and grades were used across different entities predicted to have similar survival. However, in WHO CNS5, the switch to within-tumor-type grading has been used to many tumor types (Tran and Bielle, 2022). Moreover, based on the recommendations of the 2019 cIMPACT-NOW Utrecht meeting, WHO CNS5 has simplified tumor nomenclature for better clinical utility, for example, “anaplastic astrocytoma” and “anaplastic oligodendroglioma” are no longer used; instead, such tumors are simply referred to as grade 3. Several new tumor types and subtypes are introduced in the 2021 classification because of novel diagnostic technologies, including NGS or DNA methylome profiling (Morganti et al., 2019; Wong et al., 2020). This updated WHO classification has important implications for diagnosis, management, and development of novel treatments, with the application of targeted therapies and use of combined immunological and molecular approaches (Horbinski et al., 2022). Although WHO CNS5 classification is a major advance for clinicians to choose the most tailored therapies and identify more homogeneous patient populations with the same clinical outcomes, its implementation on a routine clinical basis presents some challenges that will require real-world interaction in multidisciplinary molecular tumor board (MTB). These meetings comprise different physician figures with specialties in oncology, radiology, surgery, pathology, molecular biology, informatics, etc., which are held to discuss the multidisciplinary management of SNC patients. The roles of MTB is to try to indicate appropriate therapy based on the identified histopathological features and genetic alteration, understanding clinical and radiological treatment responses to achieve long-term survival with a good quality of patient life (Tamborero et al., 2022).

## 2 Pediatric low-grade gliomas and glioneuronal tumors (pLGG/GNTs)

### 2.1 Overview

Although relatively rare, low-grade gliomas and glioneuronal tumors account for approximately 30% of pediatric CNS tumors (Ostrom et al., 2022). Many tumor types and subtypes are included in the pLGG/GNTs group (pediatric low-grade gliomas/glioneuronal tumors), showing histological diversities that were recognized and described over years of microscopical and immunohistochemical studies. However, these tumors frequently show overlapping morphological features, and in some cases, the salient aspects may also be absent due to limited tumor sampling (Bale and Rosenblum, 2022). Over the past decades, the development of novel molecular techniques has led to revolutionary insights into the genetic drivers of these tumors (Ryall et al., 2020a). Among novel molecular diagnostics, methylome profiling is of particular interest in pLGG/GNTs (Qaddoumi et al., 2016). MAP kinase pathway alterations are almost universally present across pLGG/GNTs, even though they may occur in different forms. In fact, specific genetic alterations may have different meanings in the diagnostic algorithm of pLGG/GNTs. In this regard, fifth edition of the 2021 WHO Classification of Tumors of the Central Nervous System put together the current knowledge regarding the clinical, histopathological, immunohistochemical, and molecular features of these tumors, opening the doors for further precision in classification and treatment of these tumors (WHO Classification of Tumours Editorial Board, 2021). In the 2021 WHO, the pLGG/GNTs group has been subclassified into three different families: pediatric-type diffuse low-grade gliomas, circumscribed astrocytic gliomas, and glioneuronal and neuronal tumors (Table 1).

**Table 1:** 2021 WHO Classification of pLGG/GNTs group, subdivided into three families: pediatric-type diffuse low-grade gliomas, circumscribed astrocytic gliomas, and glioneuronal and neuronal tumors.

Pediatric-type diffuse low-grade gliomas	Circumscribed astrocytic gliomas	Glioneuronal and neuronal tumors
**Diffuse astrocytoma, MYB- or MYBL1-altered**	Pilocytic astrocytoma	Ganglioglioma
Angiocentric glioma	**High-grade astrocytoma with piloid features (HGAP)**	Gangliocytoma
**Polymorphous low-grade neuroepithelial tumor of the young (PLNTY)**	Pleomorphic xanthoastrocytoma (PXA)	Desmoplastic infantile ganglioglioma (DIG)/desmoplastic infantile astrocytoma (DIA)
**Diffuse low-grade glioma, MAPK pathway-altered**	Subependymal giant cell astrocytoma (SEGA)	Dysembryoplastic neuroepithelial tumor (DNT)
	Chordoid glioma	**Diffuse glioneuronal tumor with oligodendroglioma-like features and nuclear clusters (DGONC)**
	Astroblastoma, MN1-altered	Papillary glioneuronal tumor (PGNT)
		Rosette-forming glioneuronal tumor (RGNT)
		**Myxoid glioneuronal tumor (MGNT)**
		Diffuse leptomeningeal glioneuronal tumor (DLGNT)
		**Multinodular and vacuolating neuronal tumor (MVNT)**
		Dysplastic cerebellar gangliocytoma (Lhermitte-Duclos disease)
		Central neurocytoma
		Extraventricular neurocytoma
		Cerebellar liponeurocytoma

In bold, there are highlighted the new entities that have been included in the fifth WHO edition of CNS Tumors.

### 2.2 Pediatric-type diffuse low-grade gliomas

#### 2.2.1 Diffuse astrocytoma, *MYB*- or *MYBL1*-altered

Diffuse astrocytoma, *MYB*- or *MYBL1*-altered is a diffusely infiltrative astroglial neoplasm composed of monomorphic cells and characterized by genetic alterations regarding *MYB* or *MYBL1* genes. This new entity has been assigned to CNS WHO grade 1 (WHO Classification of Tumours Editorial Board, 2021). *MYB*-altered neoplasms are characterized by MYB overexpression, deriving from different mechanisms. *MYBL1* belongs to the same *MYB* gene family of transcriptional transactivators and, though less studied, it shows similar structure and functions. Evidence suggests that, regardless of age, *MYB*-/*MYBL1*-altered diffuse gliomas typically behave as WHO grade 1 neoplasms and are generally indolent (Wefers et al., 2020). Diffuse astrocytoma, *MYB*- or *MYBL1*-altered is rare, accounting for only 2% of pediatric low-grade gliomas (Ryall et al., 2020a). To date, the largest series reported a median age of 29 years, with a wide range from 4 to 50 years and a male preponderance (Wefers et al., 2020), even though other series showed no clear sex predilection (Tatevossian et al., 2010; Zhang et al., 2013). Most commonly, the tumor is located in the cerebral hemispheres, preferentially in the temporal lobe (42.5% of the cases) (Slegers and Blumcke, 2020). Rarely, it may also occur in the brainstem (Ryall et al., 2020a). This tumor is part of the wide group of LEATS (long-term epilepsy-associated tumors) (Slegers and Blumcke, 2020; Wefers et al., 2020). At the MRI, the tumor appears mostly in a well-defined way but may also have, at least focally, a diffuse growth pattern (Chiang et al., 2019; Wefers et al., 2020). Histologically, diffuse astrocytoma, *MYB*- or *MYBL1*-altered typically shows low-to-moderate cellularity and is composed of well-differentiated neoplastic astrocytes with small, round-to-ovoid nuclei, diffusely permeating neuropil (Chiang et al., 2019; Wefers et al., 2020). Immunohistochemically, tumor cells reveal positivity for GFAP only, while they are negative for MAP2, OLIG2, and CD34 (Wefers et al., 2020). Molecular analysis is mandatory to define tumors with compatible features as “diffuse astrocytoma, *MYB*- or *MYBL1*-altered.” FISH may be useful to demonstrate rearrangements of *MYB* or *MYBL1* genes, but sequencing allows to determine the nature of the fusion between *MYB* or *MYBL1* and a partner gene (most frequently *PCDHGA1*, *MMP16,* and *MAML2*). *QKI* has been rarely observed as partner of *MYB* in this entity, while *MYB*::*QKI* fusion is typical of angiocentric glioma (Zhang et al., 2013; Qaddoumi et al., 2016; Chiang et al., 2019; Wefers et al., 2020), which represents the most difficult differential diagnosis. However, almost all angiocentric glioma show *MYB* rearrangements and, most frequently, a *MYB*::*QKI* fusion. Nevertheless, it is of greater importance to distinguish these diffuse low-grade gliomas from other IDH-mutant or IDH-wild-type diffuse astrocytic gliomas, considering the different biological behavior and therapeutic approach. Diffuse astrocytoma, *MYB*- or *MYBL1*-altered shows a benign clinical behavior, even though the available outcome data are limited due to its rarity. The majority of cases had no evidence of disease or stable disease after long-term follow-up. In some cases, recurrence may occur, which seems to be more likely in patients who did not receive an initial gross resection (Chiang et al., 2019; Ryall et al., 2020a). Moreover, approximately 90% of patients, presenting with epilepsy, became seizure-free after resection. However, the remainder showed a reduction in seizure frequency after surgery (Wefers et al., 2020; Alzoubi et al., 2023).

#### 2.2.2 Angiocentric glioma

Angiocentric glioma is a diffuse glioma characterized by thin, cytologically bland, bipolar cells that aggregate in perivascular spaces. Almost all angiocentric gliomas have MYB alterations, with the most frequent rearrangement being represented by *MYB*::*QKI* fusion. This tumor has an indolent behavior and is assigned to CNS WHO grade 1. Angiocentric glioma was first described by Wang et al. (2005). In 2021, WHO has been reclassified as a form of pediatric-type low-grade diffuse gliomas (Fabbri et al., 2022; Kurokawa et al., 2022a). The epidemiological data regarding this entity are limited by its exceptional rarity (Ampie et al., 2016). Typically, angiocentric glioma presents as a supratentorial tumor, though in some cases, the brainstem has been reported. The most common clinical presentation is represented by long-term and drug-resistant epilepsy and is included in the LEAT group (Ampie et al., 2016; Kurokawa et al., 2022a). Histologically, angiocentric gliomas are composed of monomorphic, bipolar, spindle cells, with an infiltrative appearance, and they tend to show a perivascular arrangement (Wang et al., 2005). Rare cases with high mitotic activity (Miyata et al., 2012) or anaplastic transformation have been reported, but the clinical significance is unclear (McCracken et al., 2016). Tumor cells are GFAP-positive but negative for OLIG2 and neuronal markers. EMA usually reveals a dot-like or ring-like (i.e., microlumina) positivity, which is similar to ependymomas (Wang et al., 2005; Ni et al., 2015). The diagnosis of angiocentric glioma does not mandatorily require the demonstration of *MYB* rearrangements. Rare cases with co-occurring *BRAF* p.V600E mutation have been reported (Qaddoumi et al., 2016). *MYB* alterations are in common with diffuse astrocytoma, *MYB*- or *MYBL1*-altered, which represents the closer differential diagnosis and, in rare cases, may also harbor the hallmark fusion of angiocentric glioma. Angiocentric gliomas are biologically indolent, and gross total resection is usually curative (Ampie et al., 2016).

#### 2.2.3 Polymorphous low-grade neuroepithelial tumor of the young

Polymorphous low-grade neuroepithelial tumor of the young (PLNTY) comprise a group of neoplasms with variable morphology but characterized by indolent behavior, diffuse growth pattern, oligodendroglioma-like elements, calcifications, CD34 expression, and genetic alterations activating MAPK signaling. This tumor has been first described in 2017 by Huse et al. and included as a new entity in the 2021 classification with a CNS WHO grade 1 (Huse et al., 2017; WHO Classification of Tumours Editorial Board, 2021). Most commonly, PLNTY regards children, adolescents, and young adults, but it has been reported in a wide range of age (Huse et al., 2017; Riva et al., 2018). PLNTY is a supratentorial tumor, and it is typically characterized by solid and cystic components with dense calcification (Johnson et al., 2019; Chen Y. et al., 2020). At microscopic examination, PLNTY may be characterized by intratumoral heterogeneous morphology. An oligodendroglioma-like component is usually present, but cells may vary from uniformly rounded with perinuclear halos to spindled elements and may exhibit also nuclear pleomorphism and intranuclear pseudoinclusions. Calcifications are typically observable and are usually confluent (Huse et al., 2017). Immunohistochemically, tumor cells express glial markers (i.e., GFAP and OLIG2) and CD34, which may be patchy or diffuse and may also display non-neoplastic ramified neural elements in the associated cerebral cortex. In some cases, immunohistochemistry may show positivity for BRAFV600E (Huse et al., 2017; Johnson et al., 2019; Chen Y. et al., 2020). In fact, PLNTYs are characterized by the MAPK pathway activating alterations, whose demonstration is mandatory. *BRAF* p.V600E represents the most common genetic mutation, but fusions regarding *FGFR2* and *FGFR3* genes are also encountered. Based on the current literature, the *FGFR2*::*CTNNA3* fusion seems to be exclusive of this tumor even though present only in some cases (Bale, 2020). *FGFR3*::*TACC3* fusion, usually associated with a rare subtype of adult-type diffuse glioma, IDH-wildtype, has also been reported in a single case of PLNTY (Chen Y. et al., 2020). However, some features of this case may suggest some doubts in classifying it as a PLNTY: adult age, histological, and biological malignant transformation, co-occurring alterations in TP53, ATRX, PTEN *TEK,* and *RB1* genes. Additionally, the DNA methylation profile was not assessed for this case; therefore, we do not know if it would have shown the characteristic methylation signature of PLNTYs. For these reasons, pathologists and oncologists must always remember that *BRAF* and *FGFR3* alterations are not specific of PLNTYs but may also be encountered in high-grade gliomas, showing PLNTY-like morphological features (Bielle et al., 2018). Hence, further studies are needed for a complete understanding of the clinico-pathological significance of *FGFR3*::*TACC3* fusion and a better characterization of diffuse gliomas, harboring this peculiar rearrangement. For rare cases of PLNTY with recurrence or less favorable prognosis, the presence of specific MAPK-signaling alterations may suggest possible future applications of target treatments, paving the way for personalized therapy in pLGGs (Cipri et al., 2023).

#### 2.2.4 Diffuse low-grade glioma, MAPK pathway-altered

Diffuse low-grade glioma, MAPK pathway-altered is a generic category that includes a group of gliomas showing infiltrative growth pattern and being composed by bland cells with astrocytic, oligodendroglial, or mixed morphology. These tumors are characterized by pathogenic MAPK pathway alterations, such as *BRAF* p.V600E mutations or *FGFR1* alterations, in the form of *FGFR1* internal tandem duplication (ITD), tyrosine kinase domain (TKD) mutation, or fusion gene (WHO Classification of Tumours Editorial Board, 2021). This entity typically occurs in children, but epidemiological data are limited by its rarity (Ryall et al., 2020a). The localization is variable through the craniospinal axis, although it tends to privilege the cerebral hemispheres and, interestingly, seems to show site-specific genetic alterations (Ryall et al., 2020a). Histologically, MAPK pathway-altered diffuse low-grade gliomas are composed of mildly atypical glial cells infiltrating normal brain parenchyma, which may only show a moderately higher cell density. Morphological aspects may vary on the basis of the pathogenic MAPK-pathway genetic alteration. Instead, *FGFR1*-altered tumors classically show oligodendroglial-like morphology. These tumors may occasionally have a vaguely nodular architecture. Interestingly, these two entities, belonging to two different families (pediatric-type diffuse low-grade gliomas and glioneuronal tumors, respectively), share the same genetic alterations involving the *FGFR1* gene (Qaddoumi et al., 2016; Ryall et al., 2020a). *FGFR1* alterations may also be shared with other glioneuronal tumors, such as rosette-forming glioneuronal tumor (RGNT) and extraventricular neurocytoma, or with pilocytic astrocytoma (i.e., a circumscribed glioma). Comprehensively, we may refer to these as “*FGFR1*-altered low-grade neuroepithelial tumors,” having a common pathogenic genetic alteration, but leading to clinico-pathological entities that differ on the basis of tumor location, histologic features (even though with some overlap), accompanying genetic alterations, and epigenetic signature (Lucas et al., 2020). Because of the limited data regarding prognosis, a CNS WHO grade has not been assigned yet. However, it seems to have a better outcome when compared with CNS WHO grade 2 diffuse gliomas, but prognosis may depend on location, histology, and molecular alterations. In fact, the identification of specific alterations may provide important prognostic information and be predictive of therapeutic response to novel MAPK pathway-targeted therapies. Specifically, *FGFR1*-altered tumors may have beneficial effects from MEK inhibitor therapy (Cipri et al., 2023). Conversely, *BRAF* p.V600E-mutant tumors may respond to the administration of BRAF inhibitors (Hargrave et al., 2019). Furthermore, MEK inhibitors may also be useful for rare cases of diffuse low-grade glioma, MAPK pathway-altered, showing non-canonical *BRAF* mutations or other MAPK pathway-related gene alterations (Cipri et al., 2023).

### 2.3 Circumscribed astrocytic gliomas

The term circumscribed refers to the growth pattern, which is opposed to the “diffuse” tumors. They include pilocytic astrocytoma, high-grade astrocytoma with piloid features (HGAP), pleomorphic xanthoastrocytoma (PXA), subependymal giant cell astrocytoma (SEGA), chordoid glioma, astroblastoma, and MN1-altered (Table 1).

#### 2.3.1 Pilocytic astrocytoma (PA)

PA is a low-grade astrocytic tumor (CNS WHO grade 1) characterized by MAPK pathway alterations (typically, *KIAA1549*::*BRAF* gene fusion). It represents the 5% of pediatric brain tumors, arising during the first two decades of life. In children, it is located most commonly in the cerebellum, but the whole neuraxis and the midline structures could be involved (Bartek et al., 2020). Clinical manifestations are due to mass effect or increased intracranial pressure; in infant, primary dissemination is common (Perilongo et al., 1997). On imaging, it appears as a well-circumscribed lesion with a cystic and a solid component; the latter is hyperintense on T2, and the cystic wall has a variable contrast enhancement (Kornreich et al., 2001). On histology, they present as a low-to-moderately cellular tumor, composed of cells with a wide range of aspects as piloid features, oligodendrocyte-like cells, and multinucleated cells with nuclear clusters. Hyperchromatic and pleomorphic nuclei are sometimes present, but mitotic figures are uncommon. In a few cases, there is a high mitotic rate, which could indicate aggressive behavior (Rodriguez et al., 2010). Eosinophilic granular bodies and rosenthal fibers are frequent. Calcifications, hyalinized arteries, hemorrhages, and myxoid background with microcystic changes are common (Collins et al., 2015). The oligodendrocyte-like pattern may be linked to *FGFR1* mutations (Ryall et al., 2020b). PA express GFAP, S100, and OLIG2; synaptophysin is also frequently positive. IDH1 p.R132H expression and the H3 p.K28M (K27M) stain are negative. The Ki-67 index is usually low, and only focal increase may occur.

##### 2.3.1.1 Subtypes

- Pilomyxoid astrocytoma: It is an infantile tumor that develops in the hypothalamic/chiasmatic area, has a worse prognosis than a standard pilocytic astrocytoma, and has a tendency to spread throughout the CSF fluid (Jeon et al., 2008). A diffusely myxoid background is the hallmark of this subtype, while Rosenthal fibers and eosinophilic granular bodies are often absent (Alkonyi et al., 2015).
- Pilocytic astrocytoma with histological features of anaplasia: It presents the same morphological features of pilocytic astrocytoma, but with vigorous mitotic activity and sometimes necrosis and/or anaplasia. Anaplastic alterations could be observed at either the initial diagnosis or recurrence. Necrosis, subtotal resection, alternative telomere lengthening, and ATRX deletion are linked to poorer overall survival (Rodriguez et al., 2019). The molecular alterations in this subtype are similar to PA, but this tumor may sometimes show a specific methylome signature known as “DNA methylation class anaplastic astrocytoma with piloid features” (Reinhardt et al., 2018). Although this methylation class is more prevalent in neoplasms identified as pilocytic astrocytomas with histological anaplasia, there are still some controversial issues.

Pilocytic astrocytomas have a favorable overall survival in the majority of cases, even after numerous progressions. Radiation therapy is frequently adopted with a positive overall outcome (Nelson et al., 2019). Additionally, the altered MAPK pathway genes might give a target therapy through MEK inhibitors. However, the long-term results are still unknown. Pilomyxoid astrocytoma are known to behave more aggressively, while it is important to better classify and further determine the prognostic significance of pilocytic astrocytomas with histological anaplasia (Tihan et al., 1999).

#### 2.3.2 High-grade astrocytoma with piloid features (HGAP)

High-grade astrocytoma with piloid features (HGAP) is a high-grade astrocytic tumor histologically characterized by cells with thin fibrillary cytoplasmic process, which is suggested by the name itself (piloid). MAPK pathway gene alterations along with homozygous deletion of *CDKN2A/B* and/or ATRX mutation are distinctive of this tumor that clusters into a specific DNA methylation class. It is a rare tumor with a higher incidence in adults (Priesterbach-Ackley et al., 2020), with a median age of 40 years. Posterior fossa is the typical location of this tumor, but spinal and supratentorial regions can be also involved (Reinhardt et al., 2018). Histologically, HGAPs are mildly cellular, composed of moderately pleomorphic astrocytic cells with piloid features; glomeruloid proliferation of vessels is frequently observed. Necrosis and solid areas can be present. Rosenthal fibers and eosinophilic granular bodies are often observed. Although clinical, histologic, and molecular features may suggest the diagnosis of HGAP, DNA methylation analysis now represents one of the essential criteria for this tumor (Reinhardt et al., 2018). In fact, HGAP is a novel entity that is only defined by its methylome at present. Genes of the MAPK pathway are the most common reported, involving NF1 alteration most frequently, followed by *KIAA1549*::*BRAF* fusions and *FGFR1* mutations. *BRAF* p. V600E mutation can occur with a very low percentage. Furthermore, 80% of tumors harbor *CDKN2A/B* homozygous deletion, rarely *CDK4* amplification. Further less frequent chromosomal aberrations are partial gain of 12q and 17q, losses of 1p and 8p, and partial losses of chromosomes 14 and 19q. In 45% of cases, *ATRX* mutations have been described, leading to a loss of ATRX expression in neoplastic cells. In addition, in a small percentage of tumors, *TERT* promoter mutations have been found. Outcome data from a retrospective study showed an overall 5-year survival of approximately 50% (Reinhardt et al., 2018). No prognostic association with histological features or methylated *MGMT* promoter has been identified. A definitive CNS WHO grade has not yet been assigned.

#### 2.3.3 Pleomorphic xanthoastrocytoma (PXA)

Pleomorphic xanthoastrocytoma (PXA) is an astrocytic tumor (CNS WHO grade 2 or 3), typically harboring *BRAF* p. V600E point mutation associated with homozygous deletion of *CDKN2A* and/or *CDKN2B*. Its incidence is <1% (Ostrom et al., 2022); it arises in children and young adults with a median age of 20 years, but it may also occur in older patients (Perkins et al., 2012). The supratentorial compartment is interested in 98% of cases, and the temporal lobe is most frequently involved. Tumors located in the infratentorial compartment and spinal cord have been observed. Leptomeninges are frequently infiltrated by the tumor (Ida et al., 2015). On MRI, these tumors exhibit a cystic component and a solid component, with heterogeneous contrast enhancement. Histologically, they show a wide morphological spectrum, being composed of spindle, epithelioid, and/or multinucleated astrocytes, sometimes with a xanthomatous appearance. Intratumoral lymphocytes and eosinophilic granular bodies are frequently present. CNS WHO grade 2 tumors have a low mitotic activity (<5 mitoses/10 HPF) and a circumscribed growth pattern, while grade 3 PXAs show a brisk mitotic activity (≥ 5 mitoses/10 HPF) and may reveal, at least focally, an infiltrative pattern and anaplastic features (Vaubel et al., 2018). Classically, PXAs show a diffuse S100 positivity and focal GFAP expression, despite their astrocytic nature. Neoplastic cells can also show CD34 (Reifenberger et al., 2003) and focal neuronal markers positivity (Powell et al., 1996). Immunostain for BRAF V600E protein is observed in approximately 70% of tumors (Phillips et al., 2019). Reticulin staining is usually diffused within PXAs. The most common molecular alteration in these tumors involves MAPK pathway genes, leading to an aberrant activation of this pathway, and *BRAF* p.V600E accounts for approximately 80% (Vaubel et al., 2021). Less frequently, alterations in *NTRK1*, *NTRK2*, *NTRK3*, *RAF1*, and *NF1* genes can be detected (Vaubel et al., 2018). The contemporaneous presence of *CDKN2A* and/or *CDKN2B* homozygous deletion, detected in 90% of PXAs (Vaubel et al., 2021), and *BRAF* p.V600E mutation is highly suggestive (but not exclusive) of a PXA diagnosis (Nakajima et al., 2018). Further molecular alterations can be identified such as *TERT* promoter mutation or amplification, mainly in anaplastic tumors (Vaubel et al., 2021). The only essential criterion for the diagnosis of PXA is “an astrocytoma with pleomorphic tumour cells, including large multinucleated cells, spindle cells, xanthomatous (lipidized) cells, and eosinophilic granular bodies” (WHO Classification of Tumours Editorial Board, 2021). However, because of the wide morphological spectrum (sometimes resembling epithelioid glioblastomas, astroblastomas, gangliogliomas, or even atypical teratoid/rhabdoid tumors) and the possible absence/overlap of typical molecular alterations, DNA methylation profiling may be helpful in very challenging cases. Extent of resection is the major prognostic factor associated with recurrence. Even though PXAs mostly show a circumscribed growth pattern, they may disseminate at progression, and the recurrence is frequent (Kepes et al., 1979). Recurrent tumors and a high mitotic activity (CNS WHO grade 3) relate with survival (Ida et al., 2015); some studies showed that *TERT* promoter mutation can be associated with a more aggressive behavior (Vaubel et al., 2021). The presence of *BRAF* p.V600E mutation gives this tumor an important option for targeted therapy, predominantly in cases of incomplete resection.

#### 2.3.4 Subependymal giant cell astrocytoma (SEGA)

Subependymal giant cell astrocytoma (SEGA) is a low-grade astrocytic tumor (CNS WHO grade 1), composed of cells with ganglion-like appearance, usually located in the periventricular area. It is the most frequent brain tumor associated with tuberous sclerosis (TS), with an incidence rate of 5–15%, among these patients (Ahlsén et al., 1994). It arises during the first two decades of life, rarely after the age of 20–25 years or among infants. Lateral ventricular involvement is the typical site, followed by the third ventricle and retina. On imaging, it appears as a solid and partially calcified lesion; MRI appears heterogeneously hyperintense on the T2-weighted images with homogeneous and evident enhancement after contrast administration (Inoue et al., 1998). On histology, this tumor is composed of three elements: spindle cells with fibrillary cytoplasm, intermixed with gemistocytic-like cells, and large ganglion-like elements with vesicular nuclei (Sharma et al., 2004). The immunophenotype is quite characteristic as this tumor exhibits a diffuse positivity for S100, a variable expression of GFAP, and neuronal markers. Moreover, TTF1 nuclear expression is common (Hewer and Vajtai, 2015). The typical genetic alteration of SEGA in patients with TS is the biallelic inactivation of *TSC1* or *TSC2* genes, either by loss of heterozygosity or germline mutation (Henske et al., 1997). Other infrequent alterations comprise *BRAF* p.V600E mutation (Bongaarts et al., 2017) and mTOR pathway activation (Franz et al., 2016). Prognosis of this tumor is good when a macroscopically total resection is made, though big lesions tend to have a superior morbidity (de Ribaupierre et al., 2007). Because of the mTOR pathway activation in SEGAs, treatment with inhibitors may lead to substantial reduction in tumor size (Franz et al., 2016).

#### 2.3.5 Chordoid glioma

Chordoid glioma is a slow growing and well-circumscribed tumor (CNS WHO grade 2) characterized by *PRKCA* mutation. The incidence is <1%, occurring during the fourth or fifth decade of life with a median age of 45 years and a female predominance (Ampie et al., 2015). This tumor is typically localized to the third ventricle (Leeds et al., 2006), leading to obstructive hydrocephalus or visual field disorders as a consequence of the optic chiasm compression. On MRI, it appears as an isointense multilobulated mass on T1-weighted images, with homogeneous enhancement (Pomper et al., 2001). Microscopically, these tumors are composed of cords of epithelioid cells, which are embedded in mucoid/myxoid matrix. Lymphoplasmacytic infiltrate with Russell bodies are frequently observed. Mitotic figures are absent or very rare. The immunophenotype reflects the astrocytic nature of chordoid gliomas with a diffuse GFAP expression. Nuclear staining for TTF-1 (SPT24 clone) is frequent; the expression of S100 and EMA is variably present. Point mutation of *PRKCA* p.D463H is the hallmark alteration (Rosenberg et al., 2018); this alteration enhances proliferation of astrocytes and can represent a targetable mutation for therapy. Gross total resection (GTR) is the goal treatment for chordoid gliomas; when GTR is not applicable because of proximity to neurovascular structures, adjuvant radiotherapy can be considered (Ampie et al., 2015).

#### 2.3.6 Astroblastoma, *MN1*-altered

Astroblastoma, *MN1*-altered, is a rare circumscribed glial tumor harboring *MN1* alterations. The incidence rate is between 0.45 and 2.8%, and it occurs most frequently in women and the median age is 15 years. The supratentorial compartment is more commonly involved, although the cases of the brainstem and spinal cord have been recorded (Chen W. et al., 2020). On histology, they show the characteristic astroblastic pseudorosettes, which is composed of tumor cells anchored to a central blood vessel by eosinophilic cytoplasmic processes (Mhatre et al., 2019). Vascular and stromal sclerosis is another typical feature of this tumor. Neoplastic cells are organized in papillary or pseudopapillary structures. MN1-altered astroblastoma has no CNS WHO grade assigned. Neoplastic cells exhibit a varying expression of GFAP protein and Olig2; EMA and L1CAM expression is frequent (Mhatre et al., 2019). These tumors display a distinct DNA methylation pattern, and the characteristic molecular aberration is the MN1 rearrangement at chromosome band 22q12.1 with fusion partner genes, such as *BEND2,* and, less frequently, *CXXC5* at chromosome band Xp22.13 (Hirose et al., 2018). In the spinal cord, tumors with astroblastoma-like morphology have been described, harboring different gene fusions involving chromosome X and 22q12 breakpoint regions as EWSR1::BEND2 fusion (Lehman, 2023). Further pathogenic alterations have been identified in a subset of tumors, such as *CDKN2A* homozygous deletion (Lehman et al., 2019). The copy number profiles of MN1-altered astroblastomas variably demonstrated loss of chromosomes 22q, 14, and broad regions of X, reflecting the rearrangement processes (Lehman et al., 2019). MN1-altered astroblastomas have limited outcome data, and specific clinical, histological, or molecular characteristics do not appear to be associated with outcomes (Lehman et al., 2019). Tumors with high-grade histology are associated with recurrence, tumor progression, and poor prognosis (Bonnin and Rubinstein, 1989). Patients with MN1-altered astroblastomas have a high rate of local recurrence but good overall survival when associated with safe surgeries. Other than surgical resection, no additional prognostic factors have been identified (Tauziède-Espariat et al., 2019b). Because of the high survival rate, conservative treatment might be justified (Chen W. et al., 2020). A combination of radiotherapy and chemotherapy seems beneficial in cases where surgery is not feasible (Mhatre et al., 2019).

### 2.4 Neuronal and glioneuronal tumors

Glioneuronal tumors are rare tumors composed of both neural and glial components in different proportions. All neuronal and glioneuronal tumors are immunoreactive for neuronal cell markers, such as synaptophysin or neuron-specific enolase (NSE). However, in addition to neuronal marker positivity, only the glioneuronal subgroup of tumors are immunoreactive for markers of glial differentiation, such as glial fibrillary acidic protein (GFAP) or oligodendrocyte transcription factor 2 (Olig2). The fifth CNS WHO improves the role of molecular diagnostics in CNS tumor classification, combining them with traditional histology and immunohistochemistry (Krauze, 2021). The fifth CNS WHO comprises 14 different subtypes, including 3 new entities: multinodular and vacuolating neuronal tumor (MNVNT) which was only mentioned in the 2016 classification, diffuse glioneuronal tumor with oligodendroglioma-like features and nuclear clusters (DGONC), which is a provisional type, and myxoid glioneuronal tumor (MGT) (WHO Classification of Tumours Editorial Board, 2021). Multinodular and vacuolating neuronal tumor (MVNT) is a CSN WHO grade 1 neoplasm, arising in the temporal/frontal lobe of adult patients, with few pediatric examples. Most patients present with seizures, headache, episodic confusion, and dizziness. Histologically, MVNT shows clear hypomyelinated nodules with a fibrillary matrix, prominent vacuolar alteration, and monomorphic neuronal cells, which is haphazardly distributed or aligned along capillary vessels. Neoplastic cells are positive for OLIG2, doublecortin, and non-phosphorylated NFP and may express synaptophysin, and MAP2. Ki-67 is frequently low (<1%). CD34 expression may be observed in ramified neural elements and GFAP-positive reactive astrocytes of the adjacent cortex (Barresi et al., 2022). Molecular analyses may reveal MAPK pathway-activating abnormalities. Less commonly, they are associated with *BRAF* mutations or *FGFR2* fusions. Generally, they do not recur after gross total resection and remains stable also in the case of subtotal resection (Bale and Rosenblum, 2022). Diffuse glioneuronal tumor with oligodendroglioma-like features and nuclear clusters (DGONC) represents a new entity, which is included as a provisional type to the group of glioneuronal tumors. Its incidence is unknown but is presumed to be exceptionally rare. DGONC mainly occurs in pediatric age, with no sex predilection, and is localized in the cerebral hemispheres, mainly in the cortical or subcortical area of the temporal lobe. Histopathological features represent the hallmark of this tumor type, which are characterized by a diffuse growth of oligo-like and multinucleated cells, with nuclear clusters disposed as “pennies on a plate.” Tumor cells display diffuse positivity for OLIG-2 and synaptophysin, focal positivity for Neu-N and MAP2, and negativity for GFAP. The mitotic index is variable, and the Ki-67 labeling index can be up to 30%. The molecular hallmark of DGONC is monosomy of chromosome 14, which has been found in all the cases reported (Bale and Rosenblum, 2022). These tumors present a specific DNA methylation profile; however, if DNA methylation profiling is unavailable, morphological and immunohistochemical features may suggest the diagnosis. Due to the low number of cases with an available follow-up, DGONC was not assigned to a CNS WHO grade. To date, outcome data are only available for 26 patients, indicating a 5-year progression-free survival rate equal to 81% and 5-year overall survival rate equal to 89% (Barresi et al., 2022). Myxoid glioneuronal tumour (MGT) represents a newly introduced entity, which is located in septum pellucidum and deep periventricular white matter and classified as CNS WHO grade 1 due to its favorable outcome (Bale and Rosenblum, 2022). These are uncommon primary brain tumors with a peak incidence in the second and third decades of life. Histologically, MGT is circumscribed tumor composed of oligo-like cells immersed in a myxoid stroma. Some cases may show floating neurons and perivascular neuropil, which are similar to DNTs. Mitoses are very rare or absent, and the proliferative index is low. The oligo-like cells are immunoreactive for OLIG2, SOX10, GFAP, and MAP2 and negative for synaptophysin. Floating neurons, perivascular neuropil, and neurocytic rosettes are synaptophysin-positive. MGT shows a recurrent *PDGFRA* p.K385L/I dinucleotide somatic mutation, typically occurring in the absence of accompanying *PDGFRA* gene amplification. Outcome is good, even in the cases showing local recurrence or dissemination throughout the ventricular system (Barresi et al., 2022).

## 3 Pediatric-type diffuse high-grade gliomas

### 3.1 Overview

The tumor family defined as “pediatric-type diffuse high-grade gliomas” represents one of the main changes in the fifth WHO Classification of CNS Tumors (WHO Classification of Tumours Editorial Board, 2021; Gianno et al., 2022a). The term “pediatric-type” has been introduced to distinguish these tumors from the adult-type counterpart. In fact, compared with adult-type diffuse high-grade gliomas, these tumors present different clinico-pathological characteristics, with diverse prognostic and therapeutic implications (Annese et al., 2022; Tamma et al., 2023). The term “diffuse” reflects the growth pattern of these tumors, even if some cases of H3-wildtype and IDH-wildtype glioma and infant-type glioma may show a circumscribed growth pattern (Guerreiro Stucklin et al., 2019; Clarke et al., 2020). The term high-grade reflects both morphology and biologic behavior of these tumors, even though occasionally, especially in some cases of H3 K27-altered diffuse midline glioma, they may show a misleading low-grade morphology, not corresponding to their undoubtful aggressive behavior (Buczkowicz et al., 2014). Finally, even though defined as “gliomas” and surely belonging to this category, these tumors may sometimes show neuronal or embryonal differentiation (Andreiuolo et al., 2019a; Tauziède-Espariat et al., 2019a). Pediatric-type diffuse high-grade gliomas are subdivided into four different clinico-pathological entities: diffuse midline glioma, H3 K27-altered; diffuse hemispheric glioma, H3 G34-mutant; diffuse pediatric-type high-grade glioma, H3-wildtype and IDH-wildtype; and infant-type hemispheric glioma. All of these tumors represent novel entities, which is first included in the 2021 CNS WHO. The only exception is diffuse midline glioma, H3 K27-altered, which has been revised and renamed with respect to 2016 CNS WHO (WHO Classification of Tumours Editorial Board, 2021).

### 3.2 Diffuse midline glioma, H3 K27-altered (DMG)

Diffuse midline glioma (DMG) is an aggressive tumor occurring in the midline structures of the CNS. It is recognized as CNS WHO grade 4, due to its dismal prognosis, independently from its microscopical appearance. It is characterized by the loss of H3 K27me3 and is subdivided into three molecular subtypes: (1) DMG, H3 K27-mutant (encompassing K27M or K27I mutation in H3.3, H3.1, or H3.2); (2) DMG, with EZHIP overexpression (H3-wildtype); and 3) DMG, EGFR-mutant (including either EGFR mutation or amplification) (2021 CNS WHO). The H3 K27-mutant subtype is characterized by somatic heterozygous mutation in one of the genes encoding histone *H3* variants (in order of frequency: *H3.3, H3.1*, and *H3.2*) (Castel et al., 2015, 2018). The DMG subtype with EZHIP overexpression represents the rarest subtype. The increased expression of EZHIP protein may be assessed both by immunohistochemistry or molecular analyses (Castel et al., 2020). In both subtypes, the final result is a loss of H3 K27 trimethylation (H3 K27me3) due to the inhibition of the methyltransferase activity of EZH2, which is the catalytic subunit of PRC2. In the first subtype, this inhibitory effect is a consequence of the *H3* mutation (Bender et al., 2013; Lewis et al., 2013), whereas in the second subtype, this inhibitory effect is probably mediated by EZHIP overexpression, acting as an endogenous mimic of mutated *H3* genes (Jain et al., 2019). EGFR-mutant subtype commonly shows small in-frame insertions/duplications within exon 20, which encodes the intracellular tyrosine kinase domain (TKD). Alternatively, it may also present missense mutations in other exons. In some cases, also *EGFR* gene amplification has been reported, which may co-occur with *EGFR* mutation or in absence of it, hence it might be more precisely defined as EGFR-altered subtype. Furthermore, in this specific subtype, *EGFR* abnormalities seem to always co-occur with *H3* mutations or alternatively with EZHIP overexpression and are more frequently observable in DMG with a bithalamic or monothalamic presentation (Mondal et al., 2020; Sievers et al., 2021b). DMG is considered as a rare tumor, preferentially occurring in children. When located in the pons, DMG is also defined as “diffuse intrinsic pontine glioma” (DIPG) (Ostrom et al., 2022). Another common location is represented by the thalamus. In children, it usually presents with a bilateral thalamic involvement, while in adolescents and young adults, DMG tends to prefer a monothalamic or spinal location (Hoffman et al., 2016; Chai et al., 2020, 2023). On MRI, DMG appears as a solid mass, with diffuse infiltration of the surrounding structures. It is hypointense on T1, hyperintense on T2, and may show variable or no contrast enhancement on FLAIR (Poussaint et al., 2011; Giagnacovo et al., 2020). Histologically, DMG usually presents as a hypercellular diffusely infiltrative tumor, composed of neoplastic glial cells, showing a variegate spectrum of morphologies (Solomon et al., 2016). Mitosis, necrosis, and microvascular proliferation are frequently observable but are not essential for diagnosis. Immunohistochemically, they are usually positive for GFAP, OLIG2, and MAP2, with a variable expression. IDH1 is negative, while 50% of the cases show overexpression of p53, and 15% of cases present a loss of ATRX nuclear expression. In most of the cases, immunohistochemistry is sufficient to reach the diagnosis because it may assess the loss of H3 K27me3 expression (the cutoff is at least 80%) and H3 K27M or EZHIP positivity (Huang et al., 2018; Castel et al., 2020). In cases with loss of H3 K27me3, but not showing positivity for neither H3 K27M nor EZHIP, molecular analyses are mandatory in order to obtain a diagnosis. In rare cases, molecular analyses may reveal infrequent co-occurring alterations, such as *IDH1/2* mutations, *CDKN2A/B* homozygous deletions, *TERT* promoter mutations, and *MGMT* promoter methylation (Mackay et al., 2017, 2018). It is noteworthy to mention the possible co-occurrence of *H3* K27M and *BRAF* V600E mutations, which has been described not only in DMG (Gestrich et al., 2022) but also in glioneuronal tumors with a midline location (Nguyen et al., 2015; Pagès et al., 2017). Moreover, even though its significance still needs to be fully understood, the eventual presence of *BRAF* mutations or other MAPK pathway-related genetic alterations, such as *FGFR1* mutations, may be related to a better outcome and might also be predictive of response to *BRAF* or *MEK* inhibitors (Schüller et al., 2021). Considering the wide morphological spectrum and the possible presence of co-occurring mutations, DMG presents numerous differential diagnoses. The appropriate application of 2021 CNS WHO criteria is a fundamental aid to correctly diagnose this tumor. In difficult cases, molecular analyses may be a useful tool to correctly diagnose DMGs and identify the specific subtype. Moreover, molecular results may also be useful for predicting prognostic differences and, hopefully in the very next future, might suggest possible target therapies. Unfortunately, DMG prognosis currently remains invariably poor, with a 2-year survival rate of <10% (Mackay et al., 2017). Up-to-date, the therapeutic approach is based on surgery, frequently limited by location, radiotherapy, and chemotherapy (Vallero et al., 2023). CAR-T cells have yielded very promising preclinical and clinical results but are not yet part of the standard of care (de Billy et al., 2022; Majzner et al., 2022; Vitanza et al., 2023). Different clinical trials obtained promising results for the treatment of DMG with CAR T cells using different targetable antigens, such as GD2 (Majzner et al., 2022; Mackall, 2023), which are recently found to be highly expressed in DMG/DIPG. For these reasons, pathologists and oncologists should always try to obtain extensive information on each and every DMG case, not only for a precise diagnosis of specific tumor subtype but also for possible prognostic and therapeutic implications, widening the understanding of this rare and still lethal neoplasm.

### 3.3 Diffuse hemispheric glioma, H3 G34-mutant

Diffuse hemispheric glioma, H3 G34-mutant is a diffusely infiltrative tumor, arising in the cerebral hemispheres and assigned to CNS WHO grade 4 (WHO Classification of Tumours Editorial Board, 2021). Although classified as a glioma, it may show morphological and immunohistochemical aspects of neuronal differentiation, which is also confirmed by transcriptomic and epigenomic studies, suggesting a possible neuronal origin (Chen C. C. L. et al., 2020). The characteristic pathogenic alteration is a missense mutation of the *H3-3A* gene, resulting in a substitution of glycin 34 with an arginine (G34R) or less frequently with a valine (G34V) in the H3.3 protein (Wu et al., 2012; Korshunov et al., 2016), and to a consequent inhibition of SETD2 methyltransferase (Jain et al., 2020) and KDM2A lysine demethylase activity (Cheng et al., 2014). Studies on H3 G34-mutant cells demonstrate that differential binding of H3 K36me3 induces a transcriptional reprogramming, recapitulating that of the developing forebrain, and causes prominent upregulation of the proto-oncogene *MYCN* (Bjerke et al., 2013). Co-occurring alterations are *TP53* and *ATRX* mutations (present in 90–95% of cases), *MGMT* promoter methylation (Korshunov et al., 2016), and *PDGFRA* mutations (present in 50–70% of cases) (Chen C. C. L. et al., 2020). This tumor occurs at a median age of 15 years and is mainly located in the temporal or parietal lobe (Mackay et al., 2017). On MRI, it exhibits features comparable to those of other high-grade gliomas (Kurokawa et al., 2022b). Histologically, H3 G34-mutant diffuse hemispheric glioma typically presents as a highly cellular, infiltrative astrocytic tumor with brisk mitotic activity. Some cases may show an alternative pattern, which is morphologically similar to CNS embryonal tumors (Andreiuolo et al., 2019a). Immunohistochemically, GFAP expression may be variable. The embryonal-like variant usually expresses synaptophysin in a diffuse and strong manner. The negativity for OLIG2, together with ATRX loss of expression and p53 overexpression, is highly suggestive for this entity, though not specific. Hence, the demonstration of H3 G34 mutations is mandatory to diagnose this tumor, as specified in the 2021 CNS WHO diagnostic criteria. In the majority of the case, *H3* G34R and *H3* G34V mutations may be detected immunohistochemically by the two respective antibodies. However, false negative cases have been described (Gianno et al., 2021). Therefore, in cases with negative immunohistochemistry, but presenting the appropriate clinico-pathological context, molecular analyses are needed to demonstrate the presence of *H3* G34 mutation or address possible differential diagnoses. Molecular investigations may be also useful to stratify the prognosis in a more precise manner. In fact, better prognosis is associated with the presence of *MGMT* promoter methylation and *MUC* gene mutations, while a worse prognosis is associated with *PDGFRA* mutations and the amplification of oncogenes, such as *PDGFRA*, *EGFR*, *CDK4,* and *MDM2* (Korshunov et al., 2016; Hu et al., 2022; Vuong et al., 2022). Furthermore, the demonstration of alterations in *PDGFRA* and *MUC* genes might be potentially useful to open up new therapeutic options for these patients (Lucas et al., 2021; Hu et al., 2022).

### 3.4 Diffuse pediatric-type high-grade glioma, H3-wildtype, and IDH-wildtype

Diffuse pediatric-type high-grade glioma, H3-wildtype and IDH-wildtype (pHGG H3/IDH WT) represents a heterogeneous group of tumors, which is characterized by histological high-grade features, absence of histone H3 and IDH mutations, and aggressive biological behavior (CNS WHO grade 4) (WHO Classification of Tumours Editorial Board, 2021). The pHGG RTK1 subtype is characterized by *PDGFRA* amplifications. The pHGG RTK2 presents *EGFR* amplifications and *TERT* promoter mutations. The pHGG MYCN subtype, as its name suggests, is enriched for *MYCN* amplifications (Korshunov et al., 2017). Hence, in order to obtain a diagnosis of pHGG H3/IDH WT, the identification of *PDGFRA*, *EGFR,* or *MYCN* alterations is essential. Alternatively, this diagnosis may be obtained by demonstrating the alignment of the tumor methylation profile with the pHGG RTK1, pHGG RTK2, or pHGG MYCN subtypes. Some cases of pHGG H3/IDH WT may develop the following therapeutic radiation, or in the context of Li Fraumeni syndrome or germline mismatch repair deficiency (i.e., CMMRD or Lynch syndrome), and usually belong to the pHGG RTK1 subtype (López et al., 2019). On MRI, pHGG H3/IDH WT is similar to other high-grade gliomas, which usually appears as contrast-enhancing tumors with mass effect. Differently from other subtypes, pHGG MYCN tumors may show more specific characteristics, being better circumscribed, with slight perilesional edema and homogeneous contrast enhancement (Tauziède-Espariat et al., 2019a, 2020). At microscopical examination, pHGG H3/IDH WT may show either a glioblastoma-like or a primitive, undifferentiated morphology that may also co-exist in the same tumor. Giant cells may be variably present and might raise the suspicion of mismatch repair deficiency, which may be also assessed by immunohistochemistry. Therefore, in cases of pediatric high-grade gliomas presenting severe pleomorphism and/or giant cells, pathologists should always ask for immunohistochemical evaluation of mismatch repair (MMR) proteins (MLH1, PMS2, MSH2, and MSH6), in order to exclude a constitutional mismatch repair deficiency (CMMRD) (Guerrini-Rousseau et al., 2019). Immunohistochemically, they show at least focal positivity for glial markers, such as GFAP and/or OLIG2, even though MYCN subtype may also be completely negative for glial markers, expressing neuronal markers. As a defining feature, these tumors are always negative for IDH1 (R132H) and H3K27M antibodies and show a preserved nuclear expression of H3K27me3. The number of possible differential diagnoses is high. The application of 2021 CNS WHO diagnostic criteria is greatly helpful for managing differential diagnoses, although some critical issues may be raised (Gianno et al., 2022a). First, the characteristic genetic alterations of the three pHGG H3/IDH WT subtypes are not exclusive of this entity and may be frequently found in other CNS tumors. Moreover, in a considerable percentage of pHGG H3/IDH WT tumors, the subtype-specific genetic alterations (*PDGFRA*, *EGFR*, *TERT*, and *MYC*) are absent, and the diagnosis may be reached, only demonstrating an aligned methylation profile (Korshunov et al., 2017; Tauziède-Espariat et al., 2020). Hence, neither the absence nor the presence of these alterations alone should never suggest to certainly exclude or confirm this diagnosis, without an appropriate clinico-pathological and molecular context. At the state of the art, prognosis for these tumors remains unfavorable, with some differences as regard the three subtypes: worst for MYCN (median OS of 14 months), intermediate for RTK1 (21 months), and better for RTK2 (44 months). *PDGFRA* and *EGFR* alterations may represent potential therapeutic targets in these tumors but yet to be demonstrated and validated. Another therapeutic chance may be represented by immunotherapy, especially in the context of mismatch repair deficiency-related cases (Korshunov et al., 2017; Mackay et al., 2018).

### 3.5 Infant-type hemispheric glioma

Infant-type hemispheric glioma is a high-grade diffuse glioma arising in cerebral hemispheres during early childhood. CNS WHO grade has not been assigned for this new entity due to the lack of prospective outcome data. This tumor is characterized by receptor tyrosine kinase (RTK) fusions regarding NTRK family, *ROS1, ALK,* or *MET* (WHO Classification of Tumours Editorial Board, 2021). These fusions lead to an aberrant expression of a kinase domain, which drives tumorigenesis via the activation of PI3K and/or MAPK pathways. Generally, co-occurring genetic alterations are missing, even though *NSD1* mutations, with still uncertain significance, have been reported in rare cases (d’Amati et al., 2023b). Most of the cases occur very early in childhood, in particular during the first year of life. At histological examination, they appear highly cellular and composed of astrocytic cells, with mild-to-moderate nuclear pleomorphism. Rarely, gemistocytic elements, ganglion cells, or ependymal differentiation may be present (Olsen et al., 2015; Guerreiro Stucklin et al., 2019; Clarke et al., 2020). High mitotic activity, necrosis, and microvascular proliferation are frequently observable, even though biphasic tumors with low-grade and high-grade components have been reported (Ng et al., 2019; Valera et al., 2020). Immunostaining is scarcely useful for the dentification of gene fusions, as ALK positivity can be found only in some ALK-fused tumors, and NTRK shows high expression also in the normal brain tissue. Molecular analyses are fundamental in order to demonstrate the presence of a specific *RTK* fusion or an aligned DNA methylation profile. However, methylation profiling only recognizes a common subgroup, regardless of *RTK* fusion type, whereas the identification of these fusions is potentially useful for targeted therapeutic options (Olsen et al., 2015; Guerreiro Stucklin et al., 2019; Clarke et al., 2020). Finally, it is important to note that the identification of an *RTK* fusion in a morphologically high-grade glioma does not correspond to the diagnosis of infant-type hemispheric glioma. In fact, *RTK* rearrangements are also occasionally found in adult-type glioblastoma, IDH-wildtype, probably representing additional molecular events as a consequence of clonal evolution (Ferguson et al., 2018; Woo et al., 2020). Regarding prognosis, although data are currently limited for this new entity, the onset in early childhood is historically related to better outcomes as compared with pediatric-type diffuse high-grade gliomas, occurring in older children. Furthermore, each specific fusion seems to be associated with distinct prognosis. On the basis of data reported in a single study, ALK-rearranged tumors show the best prognosis (53.8% 5-year OS) as compared with the intermediate prognosis of NTRK-fused tumors (42.9% 5-year OS) and the poorer prognosis of tumors harboring *ROS1* alterations (25% 5-year OS) (Guerreiro Stucklin et al., 2019). However, to support these preliminary findings, prospective studies with bigger cohorts are required. The same consideration may be true regarding the effectiveness of small-molecule inhibitors that are showing promising responses in these tumors harboring RTK-activating fusions, although further studies are required for a complete validation (Drilon et al., 2017; Ziegler et al., 2018).

## 4 Ependymal tumors

### 4.1 Overview

Ependymal tumors are a heterogeneous group of neuroepithelial neoplasms arising from the progenitors of the ependymal cells, which line the inner cavities of the CNS. These are rare tumors, accounting for only 2–3% of all primary CNS neoplasms (Ostrom et al., 2022). They may arise along the whole neuroaxis, but most of the pediatric cases usually occur intracranially, whereas the spinal cord represents the preferential location among adults (McGuire et al., 2009). During the last years, significant novel molecular data allowed to identify distinct tumor subtypes, which are characterized by specific DNA methylation profile and genetic alterations. These molecular developments led to a substantially revised classification of the ependymal tumors, which are included in the 2021 WHO Classification of the CNS Tumors. The current classification, which is based on a combination of clinical, histological, immunohistochemical, and molecular features, subdivides ependymal tumors into three main groups, according to their location: supratentorial, infratentorial, and spinal.

### 4.2 Supratentorial ependymomas

Supratentorial ependymomas account for approximately 30% of all intracranial ependymomas, arising more frequently in pediatric age (Vera-Bolanos et al., 2015; Elsamadicy et al., 2020). Histologically, they show characteristic morphologic features of ependymomas, which appear similar across different anatomic sites. Perivascular pseudorosettes represent the hallmark feature and are composed of tumor cells organized around a central blood vessel. True ependymal rosettes, composed of tumor cells around an ependymal channel, are rarer to be observed. Immunohistochemically, ependymomas arising in other locations, are characterized by positivity for GFAP, negativity for OLIG2, and dot-like or ring-like cytoplasmic positivity for EMA. Regarding grading, ependymomas are classified as CNS WHO grade 2 or 3, principally on the basis of mitotic activity and independently from their location. However, there is no established cutoff, and the association between histological grading and outcome is not consistent. Supratentorial ependymomas are subclassified into *ZFTA*- and *YAP1*-fusion positive; therefore, molecular evaluation is necessary to demonstrate the presence of these entity-defining gene fusions (Andreiuolo et al., 2019b; Pagès et al., 2019). A significant part of supratentorial ependymomas do not show fusions involving *ZFTA* or *YAP1* genes and is currently classified as “Supratentorial Ependymomas, NEC” (Louis et al., 2018). In this context, a recent study reported that some supratentorial tumors preferentially occurred in pediatric age, showed ependymoma-like morphological and immunohistochemical characteristics, and characterized by recurrent fusions in *PLAGL1* genes (Sievers et al., 2021a).

#### 4.2.1 Supratentorial ependymoma, ZFTA fusion-positive

Supratentorial ependymoma, ZFTA fusion-positive, is a circumscribed ependymal tumor, which is characterized by a fusion involving *ZFTA* (formerly C11orf95) gene (WHO Classification of Tumours Editorial Board, 2021). *ZFTA* rearrangements are believed to be the principal oncogenic driver of the disease and originate from chromothriptic events on chromosome 11 (Parker et al., 2014). In most of the cases, *ZFTA* is fused with *RELA*, which encodes the p65 subunit of NF-κB transcription factor complex, leading to pathological activation of NF-κB signaling (Pietsch et al., 2014). In other cases, supratentorial ependymomas may present *ZFTA* fusions with gene partners that differ from *RELA*, such as *NCOA1/2, MAML2,* and *MN1*. These cases also show significant histopathological heterogeneity and lack pathological activation of NF-κB signaling (Tauziède-Espariat et al., 2021). ZFTA-fused supratentorial ependymomas are more common in children but may occur also in adults, and the most frequent location is represented by the frontal or parietal lobe (Lillard et al., 2019). Rare cases have also been reported, presenting an intracranial extra-axial location (Nowak et al., 2019) or a midline transtentorial involvement (Cardoni et al., 2023). Histologically, they usually show the typical features of other ependymomas, although unusual morphologies have been described, particularly for non-RELA tumors. Ependymomas with *ZFTA*::*RELA* fusion reveal cytoplasmic positivity for L1CAM and diffuse nuclear staining for p65 protein (encoded by *RELA* gene). The positivity for both or one of these two antibodies is reliable in predicting the presence of *ZFTA*::*RELA* fusions, although it always requires molecular confirmation. Instead, the negativity for both L1CAM and p65 consistently predicts the absence of *RELA* fusions. Conversely, non-RELA, ZFTA-fused tumors, usually show positivity only for L1CAM and negativity for p65. However, the molecular demonstration of *ZFTA* fusions is always mandatory for diagnosis, as reported in the 2021 CNS WHO criteria (2021 CNS WHO). Regarding prognosis, available data indicate that ZFTA fusion-positive tumors have the worst outcome. However, these studies exhibit significant variation, necessitating the use of prospective therapeutic trials to validate those findings (Pajtler et al., 2015; Figarella-Branger et al., 2016). In a series of ZFTA::RELA-fused ependymomas, homozygous deletion of *CDKN2A/2B* has been found to be an independent predictor of poorer survival (Jünger et al., 2020).

#### 4.2.2 Supratentorial ependymoma, YAP1 fusion-positive

Supratentorial ependymoma, YAP1 fusion-positive, is a circumscribed ependymal tumor, which is characterized by fusions involving *YAP1* gene (WHO Classification of Tumours Editorial Board, 2021). Most frequently, *MAMLD1* represents the fusion gene partner, although other genes may rarely be involved (Andreiuolo et al., 2019b). *YAP1*::*MAMLD1* fusion exerts oncogenic activity through the recruitment of nuclear factor I (NFI) and TEAD family members (Pajtler et al., 2019). As for ZFTA-fused ependymomas, these tumors preferentially occur in young children but are rarer. Differently from ZFTA-fused ependymomas, they are negative for both L1CAM and p65. The molecular demonstration of *YAP1* fusions is essential for diagnosis. Compared with ZFTA fusion-positive ependymomas, this entity appears to have a more favorable prognosis (Upadhyaya et al., 2019).

### 4.3 Posterior fossa ependymomas

Posterior fossa (PF) ependymomas, as suggested by the name, arise intra-axially in structures located in the posterior cranial fossa, mainly in the fourth ventricle or in the cerebellopontine angle (Witt et al., 2011). Numerous subgroups of posterior fossa ependymomas were identified through analysis of DNA methylation profiling and DNA/RNA NGS data, but two main subgroups, PFA and PFB, were reliably confirmed in independent investigations and have been included in the 2021 WHO classification (Mack et al., 2014, 2018). In most cases, immunostaining for H3 K27me3 serves as a reliable surrogate of DNA methylation profiling, being a crucial marker for the diagnostic categorization of PF ependymomas into two PFA and PFB subgroups (Panwalkar et al., 2017). As for other CNS tumors, the inability to perform appropriate immunohistochemical and/or molecular analyses prompts the addition of “NOS” (Louis et al., 2018). Posterior fossa ependymomas can be assigned CNS WHO grade 2 or 3. As for ependymomas arising in other sites, brisk mitotic activity and microvascular proliferation seem to be more reliable in defining histological grading, compared with necrosis and pleomorphism, but inconsistent results have been reported in the literature (Godfraind et al., 2012). Regarding prognosis, PFA show a poorer prognosis compared with PFB (Pajtler et al., 2015). Independently from the molecular group, the identification of a chromosome 1q gain is a reproducible indicator of adverse outcome (Kilday et al., 2012).

#### 4.3.1 Posterior fossa ependymoma, group A (PFA)

PFA is a circumscribed ependymal tumor, arising in the posterior fossa and aligning with the PFA molecular group of ependymomas. An ependymoma can be classified as PFA by immunohistochemical demonstration of H3 K27me3 loss of nuclear expression or DNA methylation profiling (WHO Classification of Tumours Editorial Board, 2021). Most commonly, PFA occur in younger children and show poorer prognosis compared with PFB (Pajtler et al., 2015; Witt et al., 2018). The oncogenesis of PFA is driven by epigenetic alterations, consisting in CpG islands hypermethylation and global DNA hypomethylation, associated with a reduction in the repressive histone mark H3 K27me3 (Mack et al., 2014). This reduction is the consequence of EZHIP overexpression, which mimics the oncohistone H3 K27M by binding to the EZH2 subunit of PRC2 complex and then inhibiting its methyltransferase activity (Hübner et al., 2019; Jain et al., 2019; Ragazzini et al., 2019). Immunohistochemistry is useful to demonstrate the loss of H3 K27me3 and also the presence of EZHIP overexpression (Antin et al., 2020; Nambirajan et al., 2021). Furthermore, since they are mutually exclusive, the demonstration of EZHIP overexpression allows to exclude the presence of *H3* K27 mutations, which are typical of DMG, H3 K27-altered, but has been rarely reported also in some PFA (Castel et al., 2020). However, the significance of *H3* K27 mutations reported in some PFA has yet to be determined (Gessi et al., 2016; Ryall et al., 2017). Remarkably, the fact that DMG and PFA share molecular features and often location, arising in neighboring regions of the brainstem and posterior fossa, suggests that certain cell populations in the developing hindbrain/posterior fossa are particularly sensitive to H3K27me3 states, and that, deregulated mechanisms of hindbrain/posteriors fossa development are fundamental to the biology of these tumors (Pun et al., 2023). To note, it has been reported that also germinomas arising in posterior fossa may show strong nuclear EZHIP positivity, associated with a loss of H3 K27me3, suggesting that the spectrum of neoplasms sharing these molecular features may be wider but strictly related to deregulated development of hindbrain/posterior fossa. In addition, isolated cases of MYC methylation class AT/RT and WNT-activated medulloblastoma have been shown to present EZHIP positivity, but this was only focal (<1% of positive tumor cells), and thus, this was not to considered as true EZHIP overexpression (>90% of positive tumor cells) (Antin et al., 2020).

#### 4.3.2 Posterior fossa ependymoma, group B (PFB)

Posterior fossa group B (PFB) ependymoma is an ependymal tumor aligned with the PFB molecular group of ependymomas. Nuclear expression of H3 K27me3 is typically retained, but it is not specific of PFB. Therefore, according to 2021 CNS WHO criteria, an ependymoma can be classified as PFB only by DNA methylation profiling (WHO Classification of Tumours Editorial Board, 2021). Different from PFA, they are more common in adults and are associated with a better prognosis (Witt et al., 2011). Preliminary data identified five molecular subgroups of PFB, with different epidemiological features: PFB-1, PFB-2, and PFB-3 are common in patients aged 25–30 years; PFB-4 occurs in younger people (median age: 15 years); and PFB-5 occurs in older people (median age: 40 years). Several cytogenetic alterations have been described in PFB, especially chromosomal aberrations, such as chromosome 6 monosomy, chromosome 18 trisomy, and loss of chromosome 22q, but the pathogenesis still remains currently unclear. However, even though biomarkers of worse prognosis are still fully elucidated, incomplete surgical removal and loss of chromosome 13q have been associated with poorer outcome in a single study (Cavalli et al., 2018).

### 4.4 Spinal ependymomas

According to 2021 WHO Classification of CNS Tumors, the spinal location has been recognized three types of ependymomas: (1) spinal ependymoma (morphologically similar to other ependymomas); (2) MYCN-amplified spinal ependymoma (characterized by MYCN-amplification and poorer prognosis); (3) myxopapillary ependymoma (identifying morphological features and usually localized in conus medullaris/filum terminale) (WHO Classification of Tumours Editorial Board, 2021).

#### 4.4.1 Spinal ependymoma

Spinal ependymoma is a circumscribed ependymal tumor, demonstrating classic histological features of ependymoma and lacking features of myxopapillary ependymoma or subependymoma. When testing is feasible, *MYCN* amplification is absent (WHO Classification of Tumours Editorial Board, 2021). The experimental inactivation of *NF2* in mice led to enhanced proliferation and decreased apoptosis of embryonal spinal cord neural progenitor cells, supporting the idea that *NF2* plays a significant role in the pathogenesis of spinal ependymomas (Garcia and Gutmann, 2014). With a median age at diagnosis ranging from 25 to 45 years, these neoplasms account for approximately 20% of primary spinal tumors (Koeller et al., 2000). As regards histopathological features, the rare tanycytic aspect, characterized by spindle cells with bipolar processes, is more frequently observable in spinal location. This morphology may mimic the histological appearance of schwannoma or pilocytic astrocytoma, representing a possible diagnostic pitfall. Immunohistochemistry may be helpful in differential diagnosis, showing typical ependymoma immunophenotype, along with SOX10 negativity (Vege et al., 2000). Frequent loss of chromosome 22q and mutations of *NF2* are characteristic alterations of spinal ependymomas, but molecular analyses are not essential for the diagnosis. According to the previously described morphological characteristics, CNS WHO grade 2 or 3 is assigned; nonetheless, CNS WHO grade 3 is uncommon in this anatomic compartment. Overall, 5–10 year survival rates of 90–100% indicate a favorable outcome (Panwalkar et al., 2017).

#### 4.4.2 Spinal ependymoma, MYCN-amplified

Spinal ependymoma, MYCN-amplified is a rare spinal ependymal tumor that has been recently characterized and included as a new entity in the 2021 WHO Classification of CNS Tumors (WHO Classification of Tumours Editorial Board, 2021). It shows a median age of 31 years, with a higher incidence in women, and is usually localized to the cervico-thoracic levels (Ghasemi et al., 2019; Swanson et al., 2019). Leptomeningeal dissemination is frequently observed at diagnosis or later during the course of the disease (Ghasemi et al., 2019; Swanson et al., 2019; Raffeld et al., 2020). This tumor shows the same morphological aspects of other ependymomas but almost always displays CNS WHO grade 3 histological features: microvascular proliferation, necrosis, and high mitotic count. *MYCN*, an oncogene belonging to the *MYC* family, encodes a transcription factor that controls neuronal development (Beltran, 2014). It plays a role in the pathogenesis of several tumors, such as medulloblastoma and neuroblastoma, but the mechanisms involving *MYCN* in ependymomas development are still unknown. *MYCN* amplification may be demonstrated as a surrogate by immunohistochemistry, showing strong and diffuse nuclear expression in these tumors. In some cases, a partial loss of H3 K27me3 has been observed (Swanson et al., 2019), but this is not constant (Ghasemi et al., 2019). Anyway, immunohistochemistry for *MYCN* may be a useful screening method in spinal ependymomas showing suspicious clinico-radiological and histopathological features, but the amplification should always be demonstrated by molecular analyses, such as FISH. Spinal ependymoma, MYCN-amplified also has a DNA methylation profile which is different from other ependymal tumors and CNS tumors with *MYCN* amplification (Raffeld et al., 2020). Compared with other spinal ependymomas, MYCN-amplified spinal ependymoma is an aggressive tumor with low progression-free and overall survival rates. Despite intensive treatments, all patients with reported follow-up data had recurrences (Ghasemi et al., 2019; Swanson et al., 2019; Raffeld et al., 2020).

#### 4.4.3 Myxopapillary ependymoma

Myxopapillary ependymoma is a circumscribed ependymal tumor, which is histologically characterized by a radial arrangement of tumor cells around blood vessels and perivascular myxoid changes. It commonly arises in the cauda equina, filum terminale, or conus medullaris and in the 2021 WHO Classification of CNS Tumors have been assigned to CNS WHO grade 2 (previously grade I, WHO 2016) (WHO Classification of Tumours Editorial Board, 2021). It may occur at all ages but is more common in adults (Bates et al., 2016). The pathogenic mechanisms of myxopapillary ependymomas are still unknown, although some recurring copy-number variations (Rogers et al., 2018) and upregulations of enzymes promoting a Warburg metabolic phenotype have been described (Mack et al., 2015). Histologically, tumor cells are usually arranged around hyalinized fibrovascular cores, forming multiple papillary structures. Deposition of myxoid material around blood vessels and microcysts is frequent (Prayson, 1997). Very rare cases of “anaplastic myxopapillary ependymomas,” showing high-grade morphological features, have been described (Lee et al., 2019). Immunohistochemistry shows positivity for GFAP, negativity for OLIG2, and absence of dot-like EMA positivity, which instead characterizes other ependymomas. Moreover, positivity for S100, CD99, CD56, and AE1/AE3 pancytokeratin may be found (Lamzabi et al., 2013). The prognosis is usually favorable and similar to conventional spinal ependymomas.

### 4.5 Subependymoma

Subependymoma is a rare ependymal tumor, with an excellent prognosis, assigned to CNS WHO grade 1 (WHO Classification of Tumours Editorial Board, 2021). It is more common in adults and sometimes discovered as incidental findings (Nguyen et al., 2017). Their typical location is fourth or lateral ventricles (Bi et al., 2015). At microscopic examination, subependymomas appear composed of small neoplastic glial cells, typically forming nuclear clusters within a fibrillary matrix and associated with microcysts and calcifications. In some cases, these tumors may be admixed with more classic ependymoma-like areas. These so called “mixed ependymoma-subependymoma” cases are considered to have a more aggressive behavior, comparable to conventional ependymomas (Rushing et al., 2007). Their immunophenotype is similar to ependymomas. Molecular analyses are usually not required for diagnosis, though they have shown interesting epigenetic results, presenting distinct site-specific DNA methylation profiles (Neumann et al., 2020). Outcome is usually excellent, with very rare recurrences, even after subtotal surgical resection. A single study reported some tumors with brainstem location, showing subependymoma morphology and H3 K27M mutations. Interestingly, these tumors seem not to be associated with the adverse outcome of DMG, although data are limited to the few cases of this study (Yao et al., 2019).

## 5 Embryonal tumors

### 5.1 Overview

Embryonal tumors of the CNS are characterized by genetic driving events, which are extremely aggressive, mainly affecting children (Sturm et al., 2016). DNA analysis and gene expression profiling allowed the identification of novel entities, leading to a reclassification of these tumors (Louis et al., 2014). One example is the diagnosis of medulloblastoma, which combines histopathological and molecular features (Taylor et al., 2012). Atypical teratoid/rhabdoid tumor (AT/RT), usually characterized by *SMARCB1* (or alternatively *SMARCA4*) inactivation, includes three genetically, epigenetically, and clinically different molecular subgroups: ATRT-TYR, ATRT-SHH, and ATRT-MYC (Ho et al., 2020). Embryonal tumor with multilayered rosettes (ETMR) typically harbors the amplification of a microRNA cluster on chromosome 19 (C19MC). In the new WHO classification, ETMR received an updated designation due to the newly discovered *DICER1* mutation in this tumor. Two newly introduced entities are FOXR2-activated CNS neuroblastoma and CNS tumor with *BCOR* internal tandem duplication (ITD) (WHO Classification of Tumours Editorial Board, 2021).

### 5.2 Medulloblastoma

Medulloblastoma is categorized in WHO CNS5 based on a combination of molecular and histological characteristics. Extensive transcriptome and DNA profile studies have led to the present molecular classification, which reflects the clinico-biological variability of this neoplasm (Ellison, 2020). Children are most commonly affected by medulloblastomas, which can occur at any age. This tumor accounts for approximately 20% of intracranial neoplasms in this age group, which is second only to high-grade gliomas (Ostrom et al., 2022). Several inherited cancer syndromes are associated with medulloblastomas (Waszak et al., 2018). A variety of germline mutations can be found in *ELP*, *SUFU, PTCH1* (naevoid basal cell carcinoma syndrome/Gorlin syndrome), *TP53*, *APC*, *PALB2*, and *BRCA2*. Medulloblastoma may grow in the fourth ventricle or be located in the cerebellar parenchyma (Blaser and Harwood-Nash, 1996), displaying symptoms and signs of elevated intracranial pressure caused by non-communicating hydrocephalus. Medulloblastomas have the ability to spread regionally, the leptomeninges, or occasionally outside the CNS. The majority of metastases are discovered adhering to the pia mater. The cerebrospinal fluid (CSF) or a hematogenous way is two possible mechanisms, mainly for SHH and non-WNT/non-SHH groups. Moreover, non-WNT/non-SHH medulloblastomas virtually always have distant CNS metastases at the time of the recurrence (Hill et al., 2020). Despite the fact that some molecular groupings and subgroups of medulloblastoma, such as WNT-activated tumors, exhibit a very good response to current therapy regimens and almost all of these individuals can be treated, all types of medulloblastomas are classified as embryonal tumors and CNS WHO grade 4. The histology is dominated by small, poorly differentiated cells with a high N:C ratio and high levels of mitotic activity and apoptosis. Architectural and cytological variation, on the other hand, classifies medulloblastomas into four histological subtypes: classic, desmoplastic/nodular, medulloblastoma with extensive nodularity, and large cell/anaplastic (Giangaspero et al., 1992; McManamy et al., 2007; Smolle et al., 2012). Such a wide range of morphological traits can be found in medulloblastoma molecular groupings. The new classification preserves the original four key molecular groups defined by the previous CNS WHO classification; wingless activated (WNT), sonic hedgehog (SHH) activated, and non-WNT/non-SHH. SHH tumors are classified based on *TP53* status in *TP53*-mutant and *TP53*-wildtype tumors (Taylor et al., 2012; Table 2). DNA methylation profiling, on the other hand, has resulted in the detection of 12 subgroups (four subgroups for SHH medulloblastoma and eight subgroups for groups 3 and 4) (Cavalli et al., 2017). This segmentation of molecular subgroups has important biological and clinical implications for prognosis and treatment options (Massimino et al., 2013; Goschzik et al., 2018). To distinguish between WNT, SHH, and non-WNT/non-SHH medulloblastomas, immunohistochemistry is even useful. The nuclear immunoreactivity for beta-catenin, which is found in the majority of malignant, helps to identify the WNT-activated group. The GAB1 and YAP1 protein immunostaining in the cytoplasm identifies the SHH-activated group. The medulloblastoma WNT and SHH groups both have cytoplasmic positivity for filamin A. Non-WNT/non-SHH tumors are immunonegative for GAB1 and YAP1 (Gianno et al., 2022b). Nevertheless, the gold standard for assessing the status of a medulloblastoma subgroup is DNA methylation profiling (Schwalbe et al., 2017). The best prognostic and predictive data come from combining morphological interpretation with molecular analysis. The level of diagnostic accuracy is further improved by incorporating information into genetic changes. To increase accuracy, additional genetic changes, such as *MYC* amplification, currently employed in the risk categorization, are incorporated into an integrated diagnosis. When a medulloblastoma develops in the context of a hereditary tumor syndrome, an integrated approach to diagnose with commentary provides a chance to focus on the clinical implications of germline. New potential treatment options derived from recent studies regarding metabolic changes during cancer progression. Indeed, MB subgroups demonstrate different gene expression, leading to dysregulated metabolic pathways (lipid metabolism, nucleotide metabolism, and oxidative phosphorylation) (REF indicate) associated with different prognosis. These contributed to metabolic clustering and further risk stratification groups. In particular, high-risk metabolic clusters comprise G3/G4 methylation subgroup, MYC-amplified. MYC amplification displayed upregulation of genes related to nucleotide metabolism and oxidative phosphorylation, making them a potential therapeutic target (Gwynne et al., 2022; Funke et al., 2023).

**Table 2:** Clinico-pathological and molecular aspects of Medulloblastoma subgroups.

	Subgroup	WNT		SHH				G3			G4		
Clinico-pathological aspects	Subtype	α	β	α	β	γ	δ	α	β	γ	α	β	γ
	Frequency	10–15%		28–30%				25–28%			40–45%		
	Anatomic location	Cerebellopontine angle/Cerebellar peduncle		Cerebellar hemisphere				Midline (filling fourth ventricle)			Midline (filling fourth ventricle)		
	Histology	Mostly classic, rarely LCA		Mostly ND, classic and LCA (less frequent)				Classic (most common), LCA			Classic and LCA (less frequent)		
	Age	6–12	>17	3–17	0–3	0–3	>17	0–10	3–17	0–10	3–17		
	Metastatic disease at diagnosis	8.6%	21.4%	20%	33%	8.9%	8.4%	43.4%	20%	39.4%	40%	40.7%	38.7%
	Prognosis (5-year survival)	97%	100%	69.8%	67.3%	88%	88.5%	66.2%	55.8%	41.8%	66.8%	75.4%	82.5%
Molecular aspects	Genetics	CTNNB1, DDX3X, KMT2D		PTCH1, TP53 KMT2D, DDX3X, MYCN ampl, BCOR, LDB1, GLI2 ampl				MYC ampl, OTX2 gain, SMARCA4, NOTCH, TGF-β			MYCN ampl, CDKN6 apml, SNCAIP duplications		
	Chromosomal abnormalities	Monosomy of chromosome 6		9q deletion; loss of 10q and 17p; gain of 3q and 9p				17q, 1q gain; loss of 5q and 10q			loss of 8, 10, 11; gain of 4, 7, 17, and 18		
	Genetic predisposition	APC (germline), most tumors lack CTNNB1 mutation		SUFU, PTCH1, TP53, PALB2, and BRCA2				PALB2 and BRCA2 (rare)			PALB2 and BRCA2 (rare)		

LCA, large cell/anaplastic; ND, nodular desmoplastic [data from Funakoshi et al. (2023)].

### 5.3 Atypical teratoid/rhabdoid tumor (AT/RT)

AT/RT is a high-grade neoplasm characterized by the ability to differentiate along three germ layer lines, making this tumor unique. AT/RT is distinguished genetically by biallelic inactivation of *SMARCB1* (known as INI1 or BAF47), less frequently, *SMARCA4* (BRG1) (Hasselblatt et al., 2014). It is assigned to grade 4 CNS WHO classification. The incidence rate of AT/RTs is 1.6% of all pediatric CNS tumors, and they can occur as familial cases in the context of rhabdoid tumor predisposition syndromes 1 (Hasselblatt et al., 2014), although there have been reports of *de novo* germline mutations (Bourdeaut et al., 2011). The median age of the patients is 20 months (Ostrom et al., 2022). Adult occurrence is uncommon (Chan et al., 2018). These tumors are located mainly in the supratentorial compartment, but the whole neuraxis can be involved. Immunohistochemistry reveals positivity for synaptophysin, EMA, and AML. Loss of INI1 or BRG1 expression represents the surrogate of the underlying gene mutations. DNA methylation analysis combined with gene expression profiling has identified three molecular subgroups: AT/RT-SHH, AT/RT-TYR, and AT/RT-MYC. These subgroups stratify patients based on their age, place of origin, and gene alteration pattern (Frühwald et al., 2020; Table 3). AT/RT-SHH is characterized by the upregulation of proteins in the SHH and Notch signaling pathways. Heterozygous *SMARCB1* point mutations are frequently observed (Ho et al., 2020). AT/RT-TYR is distinguished by an increase in the expression of proteins involved in the melanosomal system (tyrosinase), the bone morphogenetic protein (BMP) pathway, and transcription factors related to the development. The deletion of the *SMARCB1* gene is caused mostly by a mutation in one allele, and the second hit leads to a total or partial loss in the second allele of chromosome 22 (Ho et al., 2020). AT/RT-MYC expresses the *MYC* oncogene and the *HOX* cluster genes. It arises more frequently in the supratentorial compartment, although they rarely happen in the spinal cord. This group also includes the uncommon AT/RTs, affecting adults restricted to the sella (Ho et al., 2020; Broggi et al., 2022). A recent study also found a high correlation between histological patterns and molecular grouping (Zin et al., 2021). The prognosis for patients with AT/RT is often dismal. Clinical studies have revealed, however, that AT/RTs do not always result in a bad outcome. High-dose chemotherapy combined with stem cell rescue and radiation was linked to a 4-year survival rate of 43% (Reddy et al., 2020). Epigenomic landscapes of AT/RT subtypes may be associated with varied treatment response so that it may be possible to stratify patients with AT/RT (Mittal and Roberts, 2020).

**Table 3:** Clinico-pathological and genetics of AT/RT molecular subgroups [data from Federico et al. (2022)].

Molecular subgroups	Median age	Location	*SMARCB1* alterations	Involved pathway
AT/RT-SHH	2–5 years	Mainly supratentorial	Point mutations	SHH and NOTCH pathway
AT/RT-TYR	0–1 years	Mainly infratentorial	Point mutations	BMP and melanosomal pathway
AT/RT-MYC	>3 years	Mainly supratentorial	Extensive deletions	Overexpression of *MYC* gene and *HOX* cluster genes

### 5.4 Embryonal tumor with multilayered rosettes (ETMR)

ETMR is an embryonal rare malignancy with characteristic morphological features, which is characterized by a *C19MC* alteration or, less commonly, a *DICER1* mutation (CNS WHO grade 4). The median age of children affected by ETMR is less than 4 years. Three main histological arrangements can be observed: embryonal tumor with abundant neuropil and true rosettes (ETANTR), ependymoblastoma, and medulloepithelioma. On DNA methylation profile and gene expression, these three patterns cluster together. Different patterns of epithelial or mesenchymal development can be recognized (WHO Classification of Tumours Editorial Board, 2021). ETMRs exhibit widespread immunopositivity for LIN28A, which represents a very helpful marker for the identification of these tumors (Spence et al., 2014). The molecular detection of *C19MC* amplification or *DICER1* mutations is mandatory. Only ETMRs have the *C19MC* microRNA cluster mutation at 19q13.42, which is present in 90% of cases (Korshunov et al., 2010). Copy number profiling array FISH analysis is effective ways to identify *C19MC* changes. Only 5% of ETMRs do not show *C19MC* amplification, harboring *DICER1* mutations. Rare ETMRs that do not have a *DICER1* mutation or *C19MC* change should be categorized as NEC. The survival rates for ETMR are still extremely low, despite rigorous multimodal treatment.

### 5.5 CNS neuroblastoma, FOXR2-activated (CNS NB-FOXR2)

CNS neuroblastoma FOXR2-activated is a rare recently-described embryonal tumor displaying various degrees of neuroblastic/neuronal development and foci of ganglion elements and neuropil-rich stroma. It typically harbors rearrangements activating the transcription factor FOXR2 (CNS WHO grade 4). It arises in children classically within the supratentorial compartment, rarely with intraventricular location (Sturm et al., 2016). On MRI, this tumor typically manifests as a delineated mass with a cystic and a solid component, exhibiting a mild contrast enhancement (Holsten et al., 2021). Histologically, CNS NB-FOXR2 exhibits embryonal architecture-organized sheets. Homer-Wright rosettes and vascular pseudorosettes can also be observed. The immunoprofile shows a significant positivity for OLIG2. Synaptophysin is positive in regions with neurocytic/ganglionic differentiation. The majority of cases also have TTF1 overexpression (Holsten et al., 2021). CNS NB-FOXR2 is a recent addition to CNS 2021 WHO, discovered by using DNA methylation analysis, which revealed that several tumors may have belonged to distinct entities. This novel entity has chromosomal rearrangements with overexpression of *FOXR2* gene (Sturm et al., 2016; Louis et al., 2020). Next-generation sequencing is required for the discovery of *FOXR2* rearrangements, but copy-number analysis may be able to reveal changes to the *FOXR2* locus on chromosome Xp11.21. However, DNA methylation profiling greatly aids in the diagnosis of these cancers. Data on the prognosis of CNS NBFOXR2 are limited, but studies show that they have strong response to the current treatment (von Hoff et al., 2021).

### 5.6 CNS tumor with *BCOR* internal tandem duplication (ITD)

CNS tumor with *BCOR* internal tandem duplication (ITD) is a malignant CNS neoplasm that has an ITD in exon 15 of the BCOR gene. The reported patients’ median age at presentation is 3.5 years (the range is 0 to 22 years). The cerebral or cerebellar hemispheres are most frequently involved (De Lima et al., 2020). On MRI, they show a central cystic area and inhomogeneous contrast enhancement (Bremer et al., 2020). Some regions may exhibit a glioma-like appearance, and compact fascicular patterns are commonly connected with a branching capillary network. Myxoid or microcystic region is quite distinctive. Mitosis and palisading necrosis can also commonly occur. They diffusely express vimentin and CD56, while absent or sparse expression of OLIG2, GFAP, or S100 supports the diagnosis (Louis et al., 2020). Although nuclear expression of *BCOR* is a sensitive marker, it is not specific (Kao et al., 2016; Ferris et al., 2020). The molecular detection of the specific ITD is required for a conclusive diagnosis. It is possible to distinguish CNS tumors with *BCOR* ITD from other CNS tumors by DNA methylation profiling and gene expression patterns. Patients with these malignancies have low survival rates (Wen and Packer, 2021).

## 6 Mesenchymal tumors

Mesenchymal tumors of the central nervous system (CNS) are a broad group of tumors, showing different clinical, pathological, and biological features. In the CNS, mesenchymal tumors usually originate from the meninges, more rarely in the CNS parenchyma or choroid plexus. Nomenclature and histology of these neoplasms are often similar to the extra-CNS counterparts, but there are also some entities showing peculiar site-specific characteristics, part of them arising exclusively in the CNS. Meningioma represents the most frequent tumor arising from the meninges (Ostrom et al., 2022) but, in rare cases, may arise in unusual locations, such as the lung (Kemnitz et al., 1982) or head and neck (Kershisnik et al., 1993). However, meningiomas are believed to develop from arachnoid cap cells (Perry et al., 2004), whose origin is still topic of discussion whether they are mesenchymal or not. Contrarily to meningiomas, mesenchymal non-meningothelial tumors are uncommon. In the 2021 World Health Organization (WHO) Classification of CNS Tumors (WHO Classification of Tumours Editorial Board, 2021), the mesenchymal non-meningothelial tumors include those neoplasms that exclusively occur in the CNS, presenting particular histological or molecular features, or that are relatively common in the CNS with respect to other sites. These tumors are subclassified on the basis of their differentiation: fibroblastic and myofibroblastic tumors (solitary fibrous tumor), vascular tumors (hemangiomas and vascular malformations, hemangioblastoma), skeletal muscle tumors (rhabdomyosarcoma), chondrogenic tumors (mesenchymal chondrosarcoma, chondrosarcoma), notochordal tumors (chordoma), and tumors of uncertain differentiation (Meredith and Alexandrescu, 2022). Among tumors of uncertain differentiation, there are Ewing sarcoma and three recently described tumors, which have been recognized as new entities and included in the fifth edition of the WHO Classification of CNS Tumors: primary intracranial sarcoma, DICER1-mutant; CIC-rearranged sarcoma; intracranial mesenchymal tumor, FET::CREB fusion-positive. Overall, mesenchymal non-meningothelial tumors of uncertain differentiation often show variable and not specific histology and immunophenotype, making their diagnosis challenging. The application of molecular techniques allowed a better understanding of these tumors and led to the inclusion of novel entities in the 2021 WHO Classification of CNS tumors, mandatory requiring the identification of specific molecular alteration for the diagnosis. However, as demonstrated by recently reported molecular alterations in CNS tumors that are still missing an appropriate classification (d’Amati et al., 2023a), we are currently far from having fully understood the wide spectrum of morphological and molecular aspects that characterize CNS mesenchymal tumors.

## 7 Discussion

**Figure 1:**
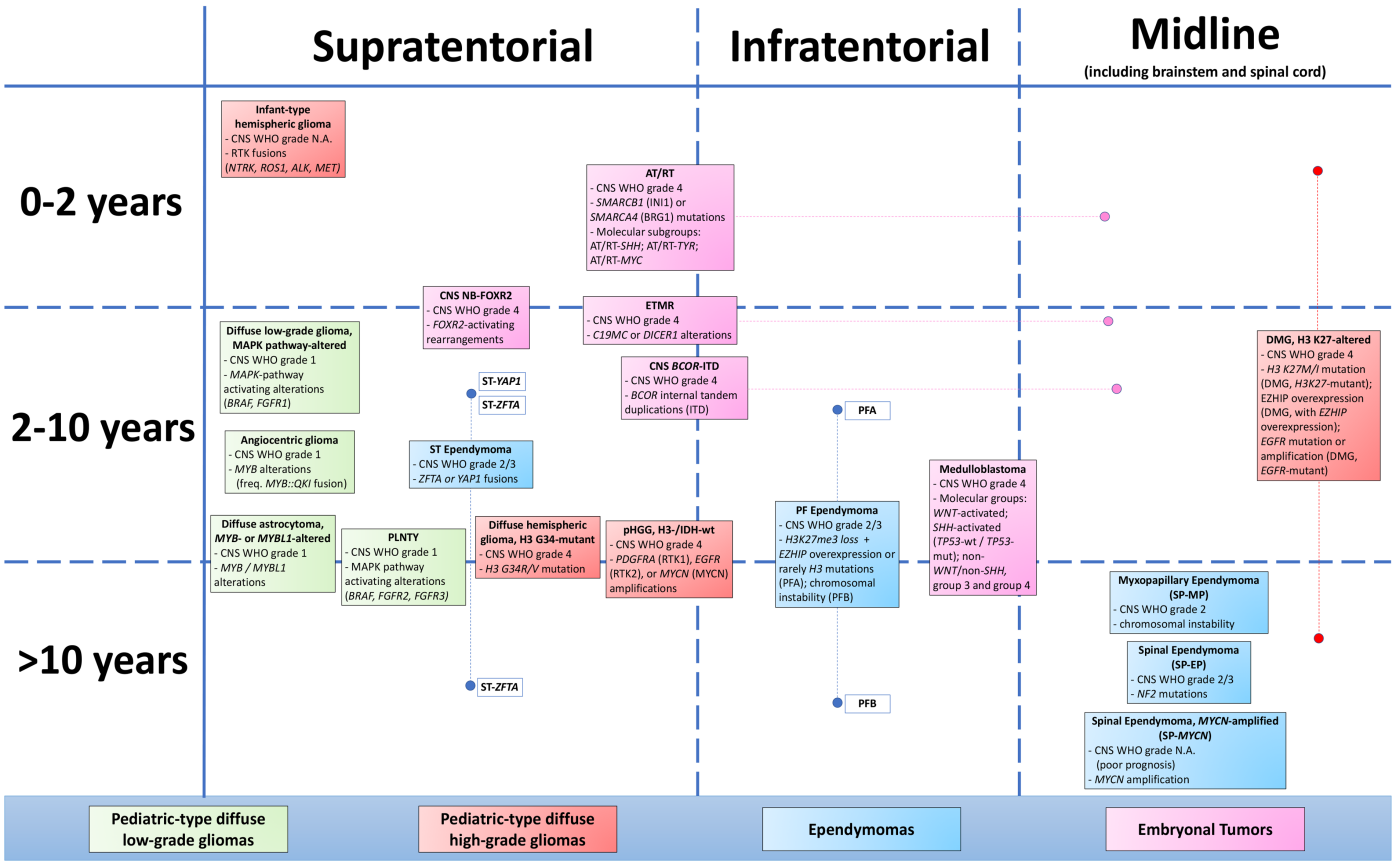
Map of pediatric CNS tumors, according to age of onset (left column) and location (first row). CNS WHO grade and relevant molecular features of each entity are presented in the figure, belonging to the groups of diffuse low-grade gliomas, diffuse high-grade gliomas, ependymomas, and embryonal tumors.

Over the last decade, molecular studies have identified an increasing number of key genetic alterations in cancers, and this has improved our knowledge and understanding of the molecular basis underlying tumor biology. Identification of these cancer-specific alterations had changed clinical approach and improved diagnosis, classification, and prognosis of CNS tumors. In the past, histologic and immunohistochemical features alone were considered for classification of CNS tumors; nowadays, the molecular findings have led to disease stratification including molecular alterations as diagnostic criteria in the fifth edition of WHO classification of tumors of the CNS. According to WHO CNS5 classification, the relevant features of some types of CNS tumors, integrating grade and molecular alterations with age and location, are presented in Figure 1. The rationale of this “molecular classification” is also related to the effective and experimental molecular therapies, targeting some cancer-specific genetic events. Additionally, molecular classification is crucial because many patients are being considered for clinical trials of targeted treatments based on the genetics described on the underlying tumor. Then, this molecular stratification has identified specific classes of entities that appear homogeneous also in their response to treatment and clinical outcomes. Regardless of this progress, further modification is needed, particularly for rare and poorly characterized tumor. These important implications for clinical practice highlight the necessity to adopt the new classification when considering therapeutic options (clinical trials, targeted therapies) and discussing prognosis.

## Author contributions

Ad’A: Conceptualization, Data curation, Writing – original draft, Writing – review & editing. LBa: Validation, Writing – original draft. SR: Conceptualization, Data curation, Validation, Writing – review & editing. AC: Conceptualization, Data curation, Validation, Writing – review & editing. LBe: Data curation, Writing – review & editing. VBa: Data curation, Validation, Writing – review & editing. ME: Data curation, Validation, Writing – review & editing. AB: Data curation, Writing – review & editing. SA: Data curation, Writing – review & editing. GM: Data curation, Writing – review & editing. GD: Data curation, Writing – review & editing. AM: Conceptualization, Data curation, Writing – review & editing. EM: Conceptualization, Data curation, Writing – review & editing. FD’A: Conceptualization, Data curation, Writing – review & editing. ES: Conceptualization, Data curation, Project administration, Resources, Supervision, Validation, Visualization, Writing – review & editing. VBi: Conceptualization, Data curation, Project administration, Resources, Supervision, Validation, Visualization, Writing – review & editing. MM: Conceptualization, Data curation, Project administration, Resources, Supervision, Validation, Visualization, Writing – review & editing. MG: Data curation, Writing – review & editing. MA: Conceptualization, Data curation, Investigation, Methodology, Project administration, Supervision, Validation, Writing – original draft, Writing – review & editing. FG: Conceptualization, Data curation, Investigation, Supervision, Writing – original draft, Writing – review & editing.
