# Corrigendum: Pediatric CNS tumors and 2021 WHO classification: what do oncologists need from pathologists?

**DOI:** 10.3389/fnmol.2024.1411360

**Published:** 2024-04-26

**Authors:** Antonio d'Amati, Lavinia Bargiacchi, Sabrina Rossi, Andrea Carai, Luca Bertero, Valeria Barresi, Maria Elena Errico, Anna Maria Buccoliero, Sofia Asioli, Gianluca Marucci, Giada Del Baldo, Angela Mastronuzzi, Evelina Miele, Federica D'Antonio, Elisabetta Schiavello, Veronica Biassoni, Maura Massimino, Marco Gessi, Manila Antonelli, Francesca Gianno

**Affiliations:** ^1^Unit of Anatomical Pathology, Department of Precision and Regenerative Medicine and Ionian Area, University of Bari “Aldo Moro”, Bari, Italy; ^2^Unit of Human Anatomy and Histology, Department of Translational Biomedicine and Neuroscience (DiBraiN), University of Bari “Aldo Moro”, Bari, Italy; ^3^Unit of Anatomical Pathology, Department of Radiology, Oncology and Anatomical Pathology, University La Sapienza, Rome, Italy; ^4^Neuropathology Unit, Fondazione Policlinico Universitario “A. Gemelli” IRCCS, Università Cattolica S. Cuore, Roma, Italy; ^5^Pathology Unit, Department of Laboratories, Bambino Gesù Children's Hospital, IRCCS, Rome, Italy; ^6^Department of Neuroscience and Neurorehabilitation, Bambino Gesù Children's Hospital, IRCCS, Rome, Italy; ^7^Pathology Unit, Department of Medical Sciences, University of Turin, Turin, Italy; ^8^Department of Diagnostics and Public Health, University of Verona, Verona, Italy; ^9^Department of Pathology, AORN Santobono Pausilipon, Pediatric Hospital, Naples, Italy; ^10^Pathology Unit, Meyer Children's Hospital IRCCS, Firenze, Italy; ^11^Department of Biomedical and Neuromotor Sciences (DIBINEM), Alma Mater Studiorum University of Bologna, Bologna, Italy; ^12^Neuropathology Unit, Fondazione IRCCS Istituto Neurologico Carlo Besta, Milan, Italy; ^13^Department of Paediatric Haematology/Oncology, IRCCS Bambino Gesù Children's Hospital, Rome, Italy; ^14^Pediatric Oncology Unit, Fondazione IRCCS Istituto Nazionale dei Tumori, Milan, Italy; ^15^IRCCS Neuromed, Pozzilli, Isernia, Italy

**Keywords:** pediatric CNS tumors, brain tumors, molecular biology, WHO classification, neuro-oncology, neuropathology

In the published article, there was an error in affiliation 10. Instead of “Pathology Unit, Meyer Children's Hospital, Firenze, Italy”, it should be “Pathology Unit, Meyer Children's Hospital IRCCS, Firenze, Italy”.

In the published article, there was an error in the author list, and authors Elisabetta Schiavello, Veronica Biassoni, and Maura Massimino were erroneously excluded. The corrected author list, affiliations, citation, copyright, and Author contributions statement appear below.

Antonio d'Amati^1, 2, 3, 4*^, Lavinia Bargiacchi^3^, Sabrina Rossi^5^, Andrea Carai^6^, Luca Bertero^7^, Valeria Barresi^8^, Maria Elena Errico^9^, Anna Maria Buccoliero^10^, Sofia Asioli^11^, Gianluca Marucci^12^, Giada Del Baldo^13^, Angela Mastronuzzi^13^, Evelina Miele^13^, Federica D'Antonio^13^, Elisabetta Schiavello^14^, Veronica Biassoni^14^, Maura Massimino^14^, Marco Gessi^4^, Manila Antonelli^3, 15*^ and Francesca Gianno^3, 15^

^1^Unit of Anatomical Pathology, Department of Precision and Regenerative Medicine and Ionian Area, University of Bari “Aldo Moro”, Bari, Italy

^2^Unit of Human Anatomy and Histology, Department of Translational Biomedicine and Neuroscience (DiBraiN), University of Bari “Aldo Moro”, Bari, Italy

^3^Unit of Anatomical Pathology, Department of Radiology, Oncology and Anatomical Pathology, University La Sapienza, Rome, Italy

^4^Neuropathology Unit, Fondazione Policlinico Universitario “A. Gemelli” IRCCS, Università Cattolica S. Cuore, Roma, Italy

^5^Pathology Unit, Department of Laboratories, Bambino Gesù Children's Hospital, IRCCS, Rome, Italy

^6^Department of Neuroscience and Neurorehabilitation, Bambino Gesù Children's Hospital, IRCCS, Rome, Italy

^7^Pathology Unit, Department of Medical Sciences, University of Turin, Turin, Italy

^8^Department of Diagnostics and Public Health, University of Verona, Verona, Italy

^9^Department of Pathology, AORN Santobono Pausilipon, Pediatric Hospital, Naples, Italy

^10^Pathology Unit, Meyer Children's Hospital IRCCS, Firenze, Italy

^11^Department of Biomedical and Neuromotor Sciences (DIBINEM), Alma Mater Studiorum University of Bologna, Bologna, Italy

^12^Neuropathology Unit, Fondazione IRCCS Istituto Neurologico Carlo Besta, Milan, Italy

^13^Department of Paediatric Haematology/Oncology, IRCCS Bambino Gesù Children's Hospital, Rome, Italy

^14^Pediatric Oncology Unit, Fondazione IRCCS Istituto Nazionale dei Tumori, Milan, Italy

^15^IRCCS Neuromed, Pozzilli, Isernia, Italy

d'Amati A, Bargiacchi L, Rossi S, Carai A, Bertero L, Barresi V, Errico ME, Buccoliero AM, Asioli S, Marucci G, Del Baldo G, Mastronuzzi A, Miele E, D'Antonio F, Schiavello E, Biassoni V, Massimino M, Gessi M, Antonelli M and Gianno F (2024) Pediatric CNS tumors and 2021 WHO classification: what do oncologists need from pathologists? *Front. Mol. Neurosci*. 17:1268038. 10.3389/fnmol.2024.1268038

© 2024 d'Amati, Bargiacchi, Rossi, Carai, Bertero, Barresi, Errico, Buccoliero, Asioli, Marucci, Del Baldo, Mastronuzzi, Miele, D'Antonio, Schiavello, Biassoni, Massimino, Gessi, Antonelli and Gianno.

Ad'A: Conceptualization, Data curation, Writing – original draft, Writing – review & editing. LBa: Validation, Writing – original draft. SR: Conceptualization, Data curation, Validation, Writing – review & editing. AC: Conceptualization, Data curation, Validation, Writing – review & editing. LBe: Data curation, Writing – review & editing. VBa: Data curation, Validation, Writing – review & editing. ME: Data curation, Validation, Writing – review & editing. AB: Data curation, Writing – review & editing. SA: Data curation, Writing – review & editing. GM: Data curation, Writing – review & editing. GD: Data curation, Writing – review & editing. AM: Conceptualization, Data curation, Writing – review & editing. EM: Conceptualization, Data curation, Writing – review & editing. FD'A: Conceptualization, Data curation, Writing – review & editing. ES: Writing – review & editing, Data curation, Supervision, Conceptualization, Project administration, Validation, Resources, Visualization. VBi: Writing – review & editing, Data curation, Conceptualization, Project administration, Validation, Resources, Visualization. MM: Writing – review & editing, Data curation, Supervision, Conceptualization, Project administration, Validation, Resources, Visualization. MG: Data curation, Writing – review & editing. MA: Conceptualization, Data curation, Investigation, Methodology, Project administration, Supervision, Validation, Writing – original draft, Writing – review & editing. FG: Conceptualization, Data curation, Investigation, Supervision, Writing – original draft, Writing – review & editing.

The authors apologize for this error and state that this does not change the scientific conclusions of the article in any way. The original article has been updated.

